# Confinement Effect and Hydrogen Species Modulation toward Enhanced Electrochemical CO_2_ Reduction to Ethanol

**DOI:** 10.34133/research.0796

**Published:** 2025-07-31

**Authors:** Yuting Zhu, Jiamin Zhu, Huizhi Li, Shuhui Li, Yue Zhai, Shao-Wen Xu, Shanshan Wu, Yuan Chen, Yafu Wang, Rui Ren, Li An, Jiangwei Zhang, Pinxian Xi, Chun-Hua Yan

**Affiliations:** ^1^ State Key Laboratory of Applied Organic Chemistry, Frontiers Science Center for Rare Isotopes, College of Chemistry and Chemical Engineering, Lanzhou University, Lanzhou, China.; ^2^ College of Energy Material and Chemistry; Inner Mongolia Advanced Research Institute; Inner Mongolia Key Laboratory of Low Carbon Catalysis; Inner Mongolia University Hohhot 010021, China; ^3^ Beijing National Laboratory for Molecular Sciences, State Key Laboratory of Rare Earth Materials Chemistry and Applications, PKU-HKU Joint Laboratory in Rare Earth Materials and Bioinorganic Chemistry, College of Chemistry and Molecular Engineering, Peking University, Beijing, China

## Abstract

The protonation process of adsorbed *CO intermediates has been widely recognized as a critical determinant governing product selectivity in electrocatalytic carbon dioxide reduction reaction (eCO_2_RR). However, the active hydrogen species and mechanism of *CO protonation in acid eCO_2_RR remain ambiguous. Particularly, the involvement of H^+^ in *CO hydrogenation is still under debate. Here, we developed a CuCl-mediated synthesis strategy integrated with rare-earth doping electronic structure engineering, which enriches intermediates and promotes adsorbed hydrogen (*H) participation in reactions, respectively. For the first time, differential electrochemical mass spectrometry (DEMS) and nuclear magnetic resonance (NMR) were employed to clarify the participation of hydrogen species in liquid and gaseous eCO_2_RR products, with isotope labeling utilized to distinguish the distribution of H^+^ and *H in the products. Experimental verification confirmed that in acidic electrolytes, the ethylene pathway was dominated by H^+^ hydrogenation, whereas the ethanol pathway incorporated contributions from both H^+^ and *H. Upon yttrium (Y) doping into Cu_2_O/CuCl, interfacial water activation was markedly enhanced, thereby enabling the provision of supplementary *H for catalytic engagement. Notably, our Y-Cu_2_O/CuCl catalyst achieves a remarkable 65.7% Faradaic efficiency for ethanol with exceptional 65-h stability at 200 mA cm^−1^. This work provides new evidence for H^+^ participation in acid eCO_2_RR, emphasizing the critical role of H_2_O activation degree in selectivity regulation, and thus offering novel insights for designing efficient acid eCO_2_RR catalysts.

## Introduction

Electrocatalytic carbon dioxide reduction reaction (ECO_2_RR) is a promising strategy for converting greenhouse gases into high-value chemicals, powered by renewable energy sources such as wind and solar, which reduces dependence on fossil fuels and enables artificial regulation of the carbon cycle [1–3]. Ethanol, producible via CO_2_ electrolysis, is in focus due to its high energy density and stable global demand, but the selectivity of ethanol still need to be enhanced for industrial application [4,5]. Understanding the pathway of eCO_2_RR is particularly important for regulating the product selectivity [6]. For typical multi-carbon products, CO_2_ is first reduced into *CO intermediates with electron transfer. Subsequently, *CO undergoes C–C coupling and a series of hydrogenations with gaining electrons, leading to eCO_2_RR products [7–9]. Various active hydrogen species play different roles in regulating product selectivity and activity of various products. In acidic eCO_2_RR hydrocarbons, there are 2 sources of hydrogen, which were from hydrogen ions in solution and water, respectively [10,11]. As for the first one, hydrogen ions will be involved in the hydrogenation process directly, while water should first be electrically reduced to *H (H_2_O + e^−^ → *H + OH^−^), which was demonstrated to be related to the degree of water activation [12]. Therefore, modulating the competitive interplay between *H and proton transfer during *CO hydrogenation is pivotal for tailoring the product selectivity in the eCO_2_RR.

Copper-based catalysts are a research hotspot due to their appropriate *CO binding affinity and excellent capability for producing multi-carbon products [13–15]. The confinement effect inherent in the porous architecture of materials has been demonstrated to effectively enrich reaction intermediates, thereby facilitating C–C coupling [16,17]. Rare earth elements are strategically employed to modulate oxygen-involving reaction pathways by their exceptional oxygen affinity [18,19]. Rational morphological engineering of Cu_2_O combined with rare-earth element doping is anticipated to enable enhanced activity and durability in the CO_2_RR.

Herein, we proposed a CuCl-mediated synthesis strategy to adjust particle size and an yttrium doping approach to promote *H participation in reactions, which modulates the active hydrogen species and improves the selectivity of ethanol. Differential electrochemical mass spectrometry (DEMS) and nuclear magnetic resonance (NMR) were employed for the first time to analyze the active hydrogen species in liquid and gas products. The preferential occurrence of the ethanol pathway is attributed to its demand for elevated adsorbed hydrogen (*H) coverage, primarily supplied through water activation. The design of yttrium-doped Cu_2_O/CuCl (Y-Cu_2_O/CuCl) catalyst was implemented to address this issue. First, by modulating the anions in the precursor salts during the synthesis, the particle size of the catalyst was increased to promote the enrichment of intermediates and inhibit the hydrogen precipitation reaction (HER). The doping of yttrium promotes water activation and increases the ratio of K**·**H_2_O, which makes our Y-Cu_2_O/CuCl exhibit a remarkable Faradaic efficiency of 65.33% for ethanol at 200 mA cm^−1^ with exceptional sustained stability exceeding 65 h.

## Results and Discussion

### Structure and properties of Cu_2_O/CuCl

**Fig. 1.**
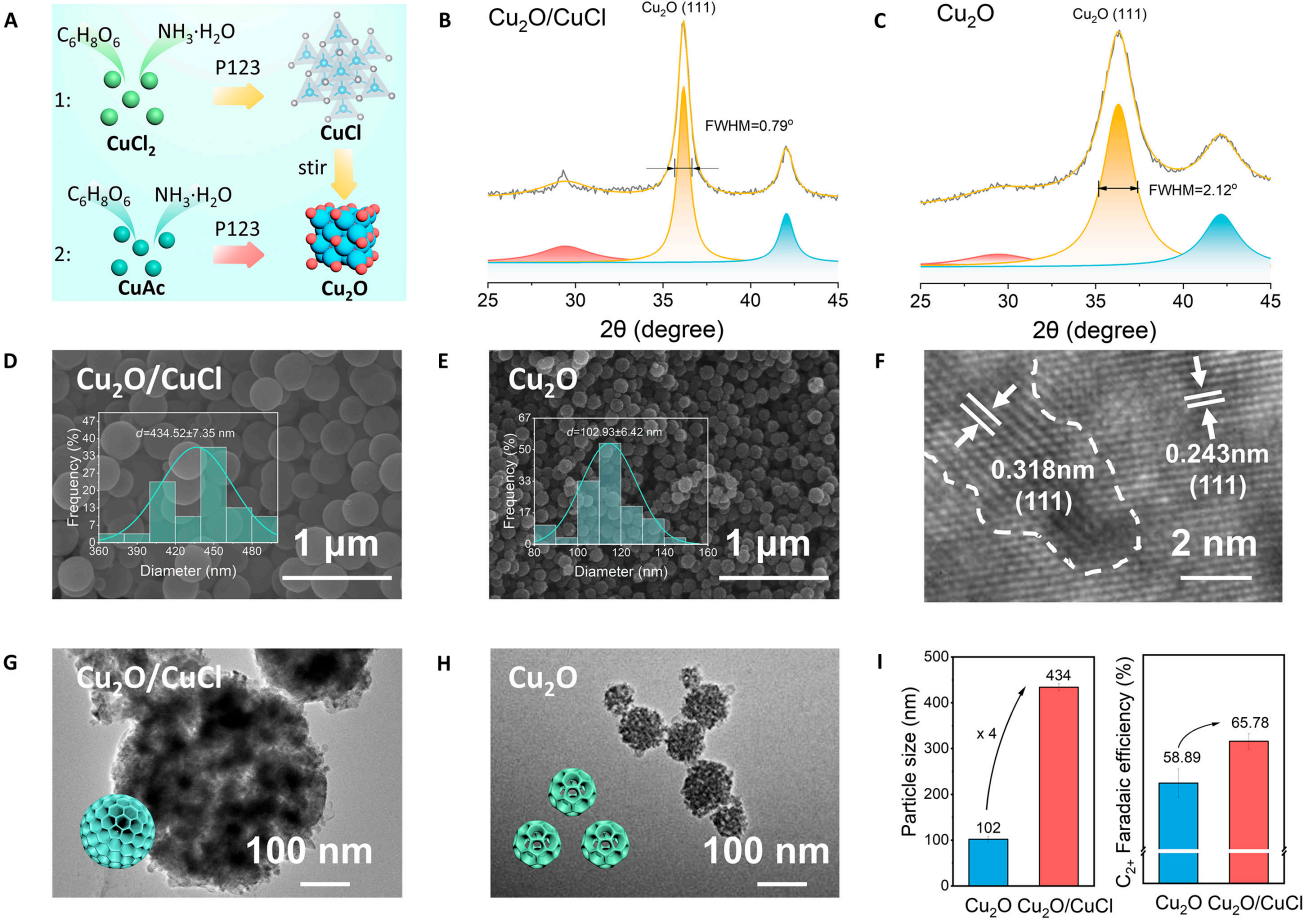
Structure and properties of CuCl-mediated Cu_2_O. (A) Simplified schematic illustration of synthesis process. (B and C) XRD pattern of (B) Cu_2_O/CuCl and (C) Cu_2_O. (D and E) SEM images of (D) Cu_2_O/CuCl and (E) Cu_2_O. (F) HRTEM image showing representative interface profiles within Cu_2_O/CuCl. (G and H) TEM images of (G) Cu_2_O/CuCl and (H) Cu_2_O. (I) Particle size and C_2+_ faradaic efficiency of Cu_2_O and Cu_2_O/CuCl.

In this work, we developed a CuCl-mediated soft-template strategy to synthesize structurally controlled Cu_2_O/CuCl nanoparticles (Fig. 1A). Specifically, ammonia was introduced into copper chloride solution to form a 6-coordinated [Cu(NH_3_)_4_(H_2_O)]^2+^ complex, as verified by ultraviolet–visible spectroscopy (Fig. S1), revealing a characteristic absorption peak at 615 nm [20,21]. Subsequent reduction with ascorbic acid produced a white nantokite phase of CuCl precipitation (PDF#1-759; Fig. S2). This CuCl precipitation transformed into the thermodynamically stable cuprite phase of Cu_2_O (PDF#1-1142; Fig. S3) after 30 min, generating the Cu_2_O/CuCl heterojunction structure designated as Cu_2_O/CuCl. When copper chloride was substituted with copper acetate, the [Cu(NH_3_)_4_(H_2_O)]^2+^ complex was similarly detected at 615 nm (Fig. S4), yet there was no CuCl precipitation that could be observed. Subsequently, the yellow Cu_2_O precipitation with nantokite phase (PDF#1-759; Fig. S5) was reduced directly by ascorbic acid. Furthermore, the Cu_2_O/CuCl heterojunction structure was confirmed by electron paramagnetic resonance (EPR). The distinct single-electron signal in the EPR spectrum demonstrated the incomplete replacement of CuCl by Cu_2_O (Fig. S6) [22]. As shown in Fig. 1B and C, x-ray diffraction (XRD) patterns revealed a marked narrowing of the full width at half maximum (FWHM) for the Cu_2_O (111) diffraction peak. Lorentzian fitting of XRD patterns for Cu_2_O and Cu_2_O/CuCl demonstrated FWHM values of 2.12 and 0.79, respectively (Fig. S7). According to Scherrer’s equation, this difference indicates a larger crystallite size in the Cu_2_O/CuCl composite.

To observe the effect of precursor salt on the size of nanoparticles, the morphology of the prepared samples was analyzed by scanning electron microscope (SEM). Both Cu_2_O and Cu_2_O/CuCl have spherical morphologies with uniform size distribution (Figs. S8 and S9). The diameters of Cu_2_O and Cu_2_O/CuCl particles were statistically analyzed using 30 randomly selected particles. A 4-fold diameter increase in Cu_2_O/CuCl (434.52 ± 7.35 nm; Fig. 1D) compared to that of pristine Cu_2_O (102.93 ± 6.42 nm; Fig. 1E) was demonstrated. This marked dimensional expansion can be attributed to the CuCl intermediate phase acting as a kinetic modulator, effectively retarding grain assemblage processes through surface coordination mechanisms.

Further structural characterization was made by transmission electron microscopy (TEM), energy-dispersive x-ray spectroscopy (EDX), and x-ray photoelectron spectroscopy (XPS). As shown in Fig. 1G and H, Cu_2_O and Cu_2_O/CuCl display the porous architecture, with Cu_2_O/CuCl exhibiting markedly enlarged nanoparticle dimensions. The interfacial lattice spacings of 0.243 and 0.318 nm in high-resolution TEM images can be corresponded to the Cu_2_O (111) plane and CuCl (111) plane, respectively, thereby validating the heterojunction interface formation (Fig. 1F). In contrast, only Cu_2_O (111) plane was detected in Cu_2_O (Fig. S10). As shown in EDX mapping (Fig. S11), Cu, Cl, and O exhibits homogeneous elemental distribution. Furthermore, XPS was employed to probe the surface electronic structure and chemical states of the catalysts. High-resolution XPS spectra of Cu 2p (Figs. S12 to S14) indicated predominant [Cu^0^, Cu^+^] and Cu^2+^ surface species, with the Cu^2+^/[Cu^0^, Cu^+^] ratio elevated in Cu_2_O/CuCl, due to the fact that Cl^−^ is a weak-field ligand and its interaction with the 3d orbitals of Cu is relatively weak, leading to easier loss of electrons from Cu. In contrast, the strong-field ligand O^2−^, with its high electronegativity, stabilizes Cu^+^ in a low-spin configuration. Mott–Schottky measurements were employed to probe the energy band structure, which further revealed conduction band positions of −0.71 V versus Ag/AgCl and −0.74 V versus Ag/AgCl for Cu_2_O and Cu_2_O/CuCl (Fig. S15), respectively. This fact suggests that enhanced reducibility in the heterostructure contributes to better performance in CO_2_RR.

The electrocatalytic performance of Cu_2_O and Cu_2_O/CuCl was evaluated in 0.5 M K_2_SO_4_ +H_2_SO_4_ (pH 3) using a flow cell. Superior C_2+_ Faradaic efficiency for Cu_2_O/CuCl (65.78% at −200 mA cm^-2^; Fig. S16) compared to Cu_2_O (58.89%) (Fig. 1I) was demonstrated. Meanwhile, the Faraday efficiency of H_2_ was obviously suppressed (Fig. S17). A strong positive correlation between nanoparticles size and C_2+_ selectivity was established, indicating that enlarged nanoparticle size facilitates intermediate enrichment.

### Structure and valence states of Y-Cu_2_O/CuCl

**Fig. 2.**
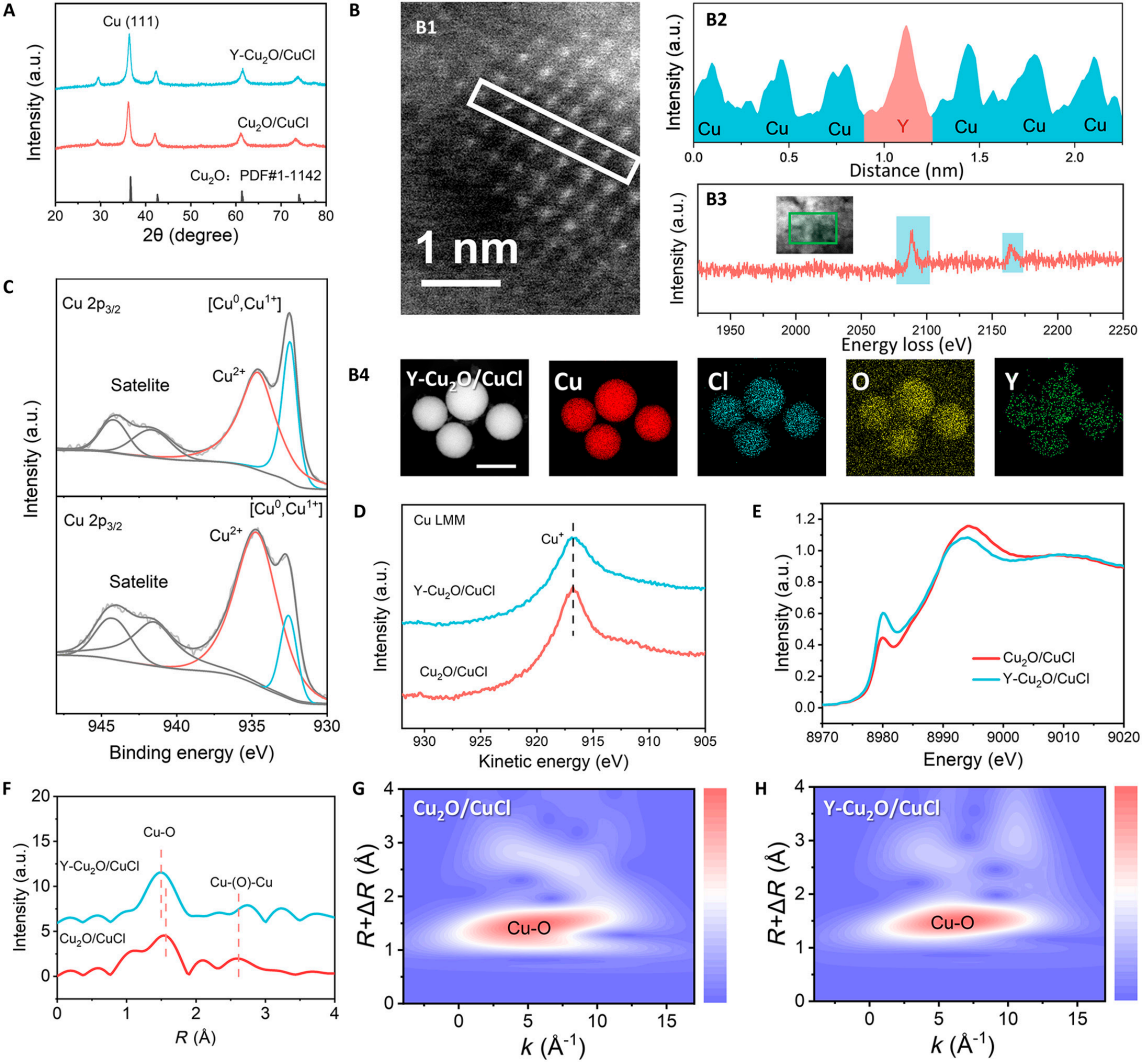
Characterization of the structure and valence states of Y-Cu_2_O/CuCl. (A) XRD patterns of Cu_2_O/CuCl and Y-Cu_2_O/CuCl. (B) HAADF-STEM of Y-Cu_2_O/CuCl at 1 nm. (B2) EDX mapping of Y-Cu_2_O/CuCl at 500 nm. (C) High-resolution XPS spectra of Cu 2p3/2 in Cu_2_O/CuCl and Y-Cu_2_O/CuCl. (D) Copper LMM Auger transition (Cu LMM) spectra. (E) Normalized Cu K-edge XANES spectra. (F) Fourier transform EXAFS spectra of Cu_2_O/CuCl and Y-Cu_2_O/CuCl. (G and H) WTs for the k3-weighted EXAFS of Cu_2_O/CuCl (G) and Y-Cu_2_O/CuCl (H).

Rare earth elements have been shown to modulate oxygen species intermediates in electrocatalytic processes [23], and we hope to exploit the inherent oxyphilicity of rare earth elements to stabilize oxygen-containing intermediates in eCO_2_RR. Y^3+^ was prioritized as the dopant of choice due to its compatible ionic radius (0.90 Å versus Cu^+^: 0.77 Å), which promotes localized lattice strain and subsequent exposure of unsaturated coordination sites. Yttrium (3%) was doped into the Cu_2_O/CuCl heterostructure for modulation, named Y-Cu_2_O/CuCl. As shown in Fig. 2A, XRD revealed that the Y-doped composite retained the primary cuprite phase (Cu_2_O, PDF:1-1142), confirming structural stability post-doping. SEM imaging exhibited uniformly distributed spherical architectures of Y-Cu_2_O/CuCl, with statistical analysis yielding an average diameter of 357.38 ± 5.43 nm (Fig. S18), indicating negligible particle size variation upon Y introduction. TEM images of Y-Cu_2_O/CuCl showed an abundant porous structure. Significantly, the existence of Cu_2_O (111) and CuCl (111) crystal planes within the composite material were further identified through high-resolution TEM (HRTEM), with the existence of heterojunction interfaces in Y-Cu_2_O/CuCl being directly confirmed by this observation (Fig. S19). Aberration-corrected scanning TEM (AC-STEM) in high-angle annular dark-field (HAADF) mode further exhibited the widespread presence of porous channels in Y-Cu_2_O/CuCl (Fig. S20). HAADF image revealed Y atoms (*Z* = 39) as localized bright spots due to their higher atomic number than Cu (*Z* = 29), consistent with substitutional doping at Cu lattice sites (Fig. 2B-B1 and Fig. 2B-B2). Figure 2B-B3 displays the electron energy loss spectroscopy (EELS) spectrum of Y-Cu_2_O/CuCl, demonstrating Y doping incorporation into the Cu_2_O lattice. Simultaneously, EDX mapping indicated the uniform distribution of Cu, Cl, O, and Y elements in Y-Cu_2_O/CuCl (Fig. 2B-B4). The actual Y content in Y-Cu_2_O/CuCl was analyzed by inductively coupled plasma optical emission spectrometry (ICP-OES), with results revealing an actual Y mass content of 0.115% in the composite (Table S1).

XPS was employed to interrogate the surface chemical states of Y-Cu_2_O/CuCl. High-resolution XPS spectra of Cu 2p_3/2_ showed dominant surface Cu^2+^ species, evidenced by satellite peaks at 940 to 945 eV (Fig. 2C) [24]. The increased Cu^2+^/[Cu^0^, Cu^+^] ratio is observed upon Y doping. The Cu 2p peak of Y-Cu_2_O/CuCl is also observed to be positively shifted by 0.2 eV, relative to Cu_2_O/CuCl. It is attributed to the decrease in the copper center charge density in the Cu–O–Y unit due to the inherent oxygenophilicity of Y attracting electrons through the oxygen bridge. To resolve Cu^+^ contributions, Auger electron spectroscopy (AES) was employed, detecting a characteristic Cu^+^ peak at 917 eV (Fig. 2D). Concurrently, the Y 3d_5/2_ peak at 156.5 to 157.5 eV confirmed Y^3+^ incorporation into the lattice, excluding metallic Y (155.3 eV) (Fig. S21).

The coordination environment and local electronic structure were investigated synergistically using x-ray absorption near-edge structure (XANES) and extended x-ray absorption fine structure (EXAFS). The pre-edge peak at 8,983 eV, which corresponds to the 1s → 3d transition accompanied by ligand-to-metal charge transfer, was analyzed to probe the coordination structure of Cu sites in XANES spectra (Fig. 2E) [25,26]. Notably, XANES showed that Y-Cu_2_O/CuCl exhibited a higher oxidation state and a highly disordered local structure compared to the pristine material. EXAFS spectra further resolved the fine coordination environment and geometric interfacial configuration of Cu (Fig. 2F). Fourier-transform EXAFS spectra of Y-Cu_2_O/CuCl exhibited 2 distinct radial distribution peaks at 1.50 and 2.61 Å, assigned to Cu–O and Cu–(O)–Cu scattering paths, respectively [27]. The Cu–O bond length is shortened from the original 1.56 to 1.50 Å, which proves that the yttrium-induced modification leads to the enhancement of Cu–O bond covalency through localized electronic structure modulation.

The coordination environment was further intuitively confirmed by wavelet transform (WT) analysis of the Cu K-edge, where strong intensity maxima were observed (Fig. 2G and H). Notably, the high-intensity region at 1.6 Å was attributed to interfacial Cu–O coordination.

**Fig. 3.**
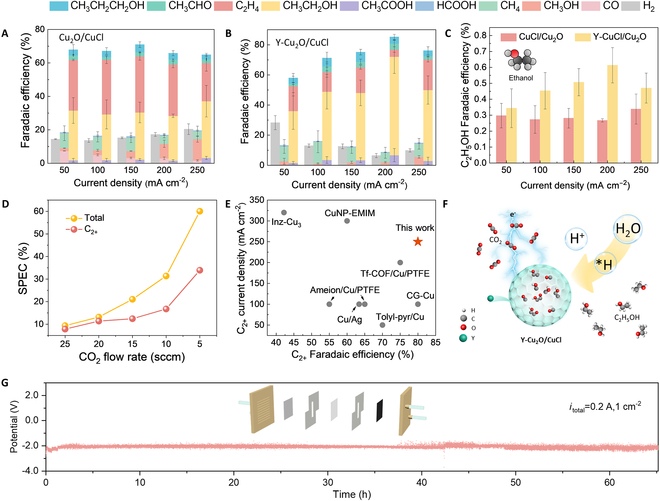
CO_2_RR performance in the gas diffusion electrode (GDE)-based flow cell. (A and B) Faradaic efficiency of (A) Cu_2_O/CuCl and (B) Y-Cu_2_O/CuCl. The error bars represent 3 independent measurements. (C) C_2_H_5_OH Faradaic efficiency of Cu_2_O/CuCl and Y-Cu_2_O/CuCl. (D) SPCE of Y-Cu_2_O/CuCl under different CO_2_ gas flow rate. (E) C_2_H_5_OH Faradaic efficiency and stability performance of Y-Cu_2_O/CuCl compared to other catalysts. (F) Simplified schematic illustration of Y-Cu_2_O/CuCl. (G) Stability test of Y-Cu_2_O/CuCl in 0.5 M K_2_SO_4_ (pH 3) under 200 mA cm^−2^.The error bars were derived from at least 3 independent measurements.

### Evaluation of the electrochemical performance

The eCO_2_RR performance of Y-Cu_2_O/CuCl and Cu_2_O/CuCl was systematically evaluated in a flow-cell configuration using 0.5 M K_2_SO_4_ + H_2_SO_4_ (pH 3) electrolyte under current densities of −50 to −250 mA cm^−2^. Product distribution showed that Y-Cu_2_O/CuCl primarily generated multicarbon compounds (ethylene, ethanol, and propanol) with minor quantities of methane, carbon monoxide, and formic acid. Notably, the Y-Cu_2_O/CuCl catalyst achieved a peak multicarbon Faradaic efficiency of 80.87 ± 0.07% at −200 mA cm^−2^ (Fig. 3A), outperforming the undoped Cu_2_O/CuCl, which had the highest Faraday efficiency of 66.43 ± 0.03% at a current density of −200 mA cm^−2^ (Fig. 3B). Product distribution demonstrated a distinct selectivity shift induced by Y doping: Y-Cu_2_O/CuCl exhibited 65.33 ± 11.07% ethanol selectivity at −200 mA cm^−2^, concurrently suppressing ethylene formation compared to the pristine Cu_2_O/CuCl (Fig. 3C). Ethanol/ethylene ratio of Cu_2_O/CuCl and Y-Cu_2_O/CuCl can clearly reveal this (Fig. S18). In addition, utilizing an acidic system minimizes carbonate formation and therefore facilitates overcoming the carbon utilization limitations observed in neutral and alkaline solutions. When the flow rate of carbon dioxide was reduced from 25 standard cubic centimeters per minute (sccm) to 5 sccm, the single-pass carbon efficiency (SPCE) of all products on Y-Cu_2_O/CuCl increased from 9.34% to 60.05% at −200 mA cm^−2^, whereas the SPCE of the C_2+_ products could reach 33.89% at 5 sccm (Fig. 3D). This is higher than the reported systems in alkaline/neutral electrolytes and highlights the advantage of the catalytic system. To elucidate the origin of enhanced activity, cyclic voltammetry (CV) curves were measured at scan rates of 10 to 50 mV s^−1^ within the non-Faradaic potential region. Y-Cu_2_O/CuCl exhibited a 2.22-fold enhancement in double-layer capacitance compared to the pristine Cu_2_O/CuCl, directly evidencing its expanded electrochemically active surface area (Fig. S22). To further understand the electrochemical behavior of Y-Cu_2_O/CuCl, in situ electrochemical impedance spectroscopy was performed. As shown in Fig. S23, the diameter of the semicircle in the mid-frequency region represents the charge transfer resistance [28], which decreased for all catalysts as the potential increased from OCP to −0.9 V versus reversible hydrogen electrode (RHE), indicating that the charge transfer resistance accelerated with the application of the potential. The spectrum of Y-Cu_2_O/CuCl has the smallest intercept with the horizontal axis, indicating that Y-Cu_2_O/CuCl has the smallest contact resistance. As evidenced above, the Y-doped Cu_2_O/CuCl modulation strategy has been demonstrated to effectively promote CO₂ electroreduction to ethanol (Fig. 3F). Long-term stability tests revealed that Y-Cu_2_O/CuCl maintained 60 h of continuous electrolysis at −200 mA cm^−2^ (Fig. 3G). Compared with published catalysts [29–36], Y-Cu_2_O/CuCl was the best catalyst in terms of ethanol selectivity and stability (Fig. 3E and Table S2).

### In situ electrochemical characterization of Y-Cu_2_O/CuCl

Typically, it was widely a consensus that catalysts will undergo irreversible surface remodeling before eCO_2_RR occurs [37,38]. The evolution of redox peaks in CV monitors surface restructuring of electrode materials during potential cycling. CV tests were carried out on our catalysts, which exhibited distinct oxidation and reduction peaks (Fig. S24). The oxidation peak located at about −0.2 V versus RHE is the reduction of Cu^+^ to Cu^0^. The reduction peak of Y-Cu_2_O/CuCl shifted somewhat to the left, which proves that Cu^+^ in Y-Cu_2_O/CuCl is more difficult to reduce.

To investigate the reason for the enhancement of ethanol selectivity by Y-Cu_2_O/CuCl, the electrochemical surface structure was investigated by in situ surface-enhanced Raman spectroscopy (SERS). Three peaks at 442, 528, and 621 cm^−1^ were observed in the lower energy region under open-circuit potential (OCP) conditions, corresponding to the vibrational signatures of Cu_2_O (Fig. S25) [39]. However, these characteristic peaks were completely suppressed when the Cu_2_O/CuCl electrode was polarized to −0.4 V versus RHE, suggesting surface reduction of Cu^+^ to metallic Cu^0^. In contrast, weak but persistent Cu_2_O signals were detected in Y-Cu_2_O/CuCl even at −0.8 V versus RHE, demonstrating Y-dopped stabilization of Cu^+^ species against electrochemical reduction. Additionally, the crystalline phase of Y-Cu_2_O/CuCl after electrolysis was confirmed by XRD, revealing that the main component remains Cu_2_O (Fig. S26).

Y-Cu_2_O/CuCl was dissolved for different times of electrolysis, and the Cl^−^ content in the material was monitored by ion chromatography (IC) after different times of electrolysis, and the Cl^−^ content in Y-Cu_2_O/CuCl remained invariant throughout prolonged operation (>60 min), demonstrating robust halogen retention post-electrochemical activation (Fig. S27).

The mass evolution of electrodes during CO_2_RR was quantitatively monitored using electrochemical quartz crystal microbalance coupled with CV [40,41]. Notably, Y-Cu_2_O/CuCl required 5 consecutive CV cycles to achieve mass equilibration, whereas pristine Cu_2_O/CuCl stabilized within 3 cycles, indicating prolonged structural reconstitution in Y-Cu_2_O/CuCl (Fig. S28).

**Fig. 4.**
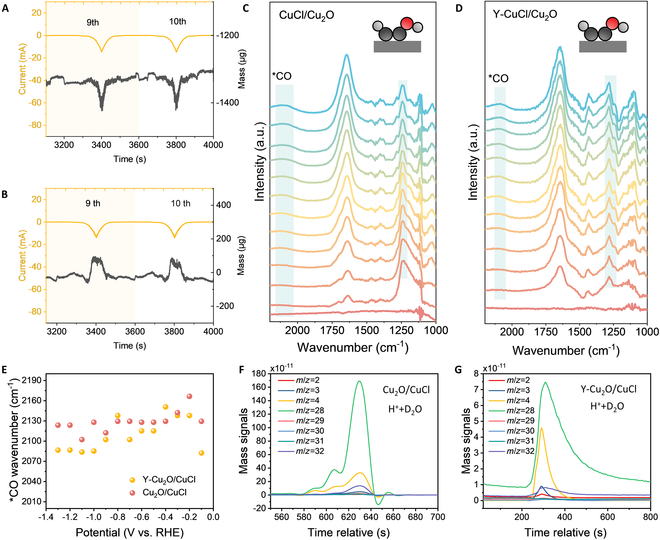
In situ electrochemical characterization of Y-Cu_2_O/CuCl. (A and B) Plot of mass change during CV scanning of Cu_2_O/CuCl (A) and Y-Cu_2_O/CuCl (B). (C and D) In situ ATR-SEIRAS spectroscopy of Cu_2_O/CuCl (C) and Y-Cu_2_O/CuCl (D). (E) Potential-dependent *CO stretching frequency. (F and G) DEMS measurements of Cu_2_O/CuCl (F) and Y-Cu_2_O/CuCl (G) in 0.5 M K_2_SO_4_ + H_2_SO_4_ (pH 3) with H_2_O.

The changes in the mass of the catalyst stabilized after reconstruction was monitored. When taking the 9th and 10th circles as examples, it was found that the mass of the Cu_2_O/CuCl electrode decreased with the increase of the applied voltage in the CV, indicating that the change in the mass of the electrode itself was greater than the change in the mass of the intermediates (Fig. 4A). Conversely, the Y-Cu_2_O/CuCl electrode exhibited a marked mass increase when voltages exceeding the overpotential were applied. This mass gain surpassed that observed in the Cu_2_O/CuCl electrode. The larger mass variation in reaction intermediates compared to the Cu_2_O/CuCl system is demonstrated in Fig. 4B. This inversion in mass evolution trends indicates that Y-Cu_2_O/CuCl enhanced intermediate adsorption capacity in CO_2_RR.

To further clarify the intermediate changes during eCO_2_RR, attenuated total reflection surface-enhanced infrared absorption spectroscopy (ATR-SEIRAS). was systematically conducted across the potential range of 0 to −1.4 V versus RHE. Firstly, Y-Cu_2_O/CuCl and Cu_2_O/CuCl exhibited a characteristic vibrational mode at about 1,250 cm^−1^, assigned to the *CH–COH intermediate (Fig. 4C and D) [42]. Compared with Cu_2_O/CuCl, a marked blue shift in the *CH–COH peak wavenumber can be observed for Y-Cu_2_O/CuCl, which results from Y doping that weakens the Cu–C bond in *CH–COH through an electron injection effect, indirectly strengthening the C–H bond and resulting in an increase in its vibrational frequency.

Furthermore, *CO adsorption signatures near 2,100 cm^−1^ were detected in both catalytic systems. Comparative analysis of peak positions revealed that the CO stretching frequency of Cu_2_O/CuCl exceeded that of the Y-doped counterpart, demonstrating a Y-induced redshift (Fig. 4E). This vibrational frequency shift indicates strengthened interactions between *CO intermediates and Y- Cu_2_O/CuCl catalytic sites.

### Exploration of the proton source in the products

CO intermediate protonation has been demonstrated by both experimental and computational studies to critically govern multicarbon product formation. However, the origin of protons for CO activation and detailed protonation pathways are still debated. Consequently, elucidating proton transfer in acidic CO_2_RR systems becomes essential, particularly for clarifying whether direct proton participation occurs.

The local pH at the electrode interface was evaluated using a rotating ring-disk electrode (RRDE) configuration. Initially, the ring disk was coated with iridium-plated iridium by electrodeposition. A standard curve was established based on the OCP differences at varying pH levels [43]. Subsequently, linear sweep voltammetry (LSV) was employed to monitor potential shifts of the pH-sensitive ring electrode, enabling the dynamic tracking of superficial proton flux during CO_2_RR (Fig. S29). It is found that Cu_2_O/CuCl consumes more protons in CO_2_RR compared to Y-Cu_2_O/CuCl. Therefore, we suggest that H^+^ is also involved in the hydrogenation process of CO_2_RR, indicating that water is not the only source of hydrogen for the process and that the involvement of H^+^ is more favorable for ethylene production.

To probe the hydrogen source in product formation, isotopic tracing was performed using H_2_SO_4_ + 0.5 M K_2_SO_4_ in D_2_O electrolyte to label deuterium in water. Initially, the gas-phase products were monitored via DEMS. Detection channels were configured to target mass/charge ratio (*m*/*z*) = 2, 3, and 4 for tracking H_2_, HD, and D_2_ species, respectively, with parallel monitoring of *m*/*z* = 28, 29, 30, 31, and 32 to detect ethylene isotopologues C_2_H_4_, C_2_H_3_D, C_2_H_2_D_2_, C_2_HD_3_, and C_2_D_4_. When H_2_SO_4_ + 0.5 M K_2_SO_4_ in H_2_O was used as the electrolyte, C_2_H_4_ with *m*/*z* = 28 was the major proportion, but a small amount of H_2_ with *m*/*z* = 2 (Fig. S30). Replacing the electrolyte as H_2_SO_4_ + 0.5 M K_2_SO_4_ + D_2_O, it was found that the C_2_H_4_ with *m*/*z* = 28 remained the predominant product, while a pronounced increase in D_2_ intensity (*m*/*z* = 4) was observed relative to HD (*m*/*z* = 3) and residual H_2_ (*m*/*z* = 2) (Fig. 4F and G). This isotopic distribution confirms preferential H^+^ utilization during ethylene hydrogenation, whereas the HER predominantly derives protons from water dissociation. It is hypothesized that H^+^ is transferred directly from the solvent water molecule to the oxygen terminus of the intermediate (e.g., the hydroxyl group of *CH–COH), leading to the deoxygenation of ethylene *CH–COH + H^+^ + e^−^ → *C–CH + H_2_O [44].

**Fig. 5.**
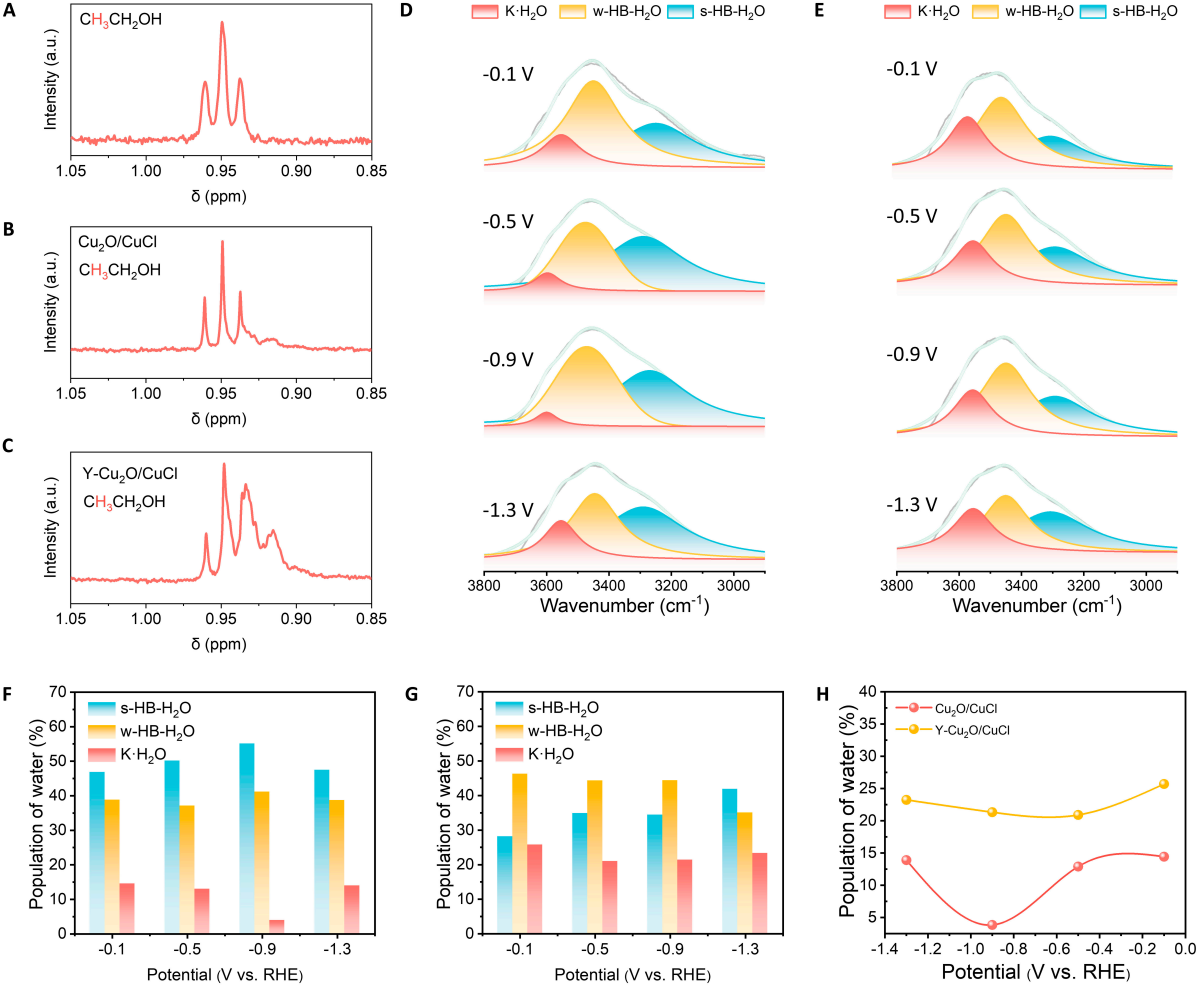
Interfacial water structure of catalysts. (A) ^1^H NMR of C_2_H_5_OH. (B and C) ^1^H NMR of C_2_H_5_OH after electrolysis Cu_2_O/CuCl (B) and Y-Cu_2_O/CuCl (C) in 0.5 M K_2_SO_4_ + H_2_SO_4_ with D_2_O. (D and E) In situ ATR-SEIRAS spectra of interfacial water on Cu_2_O/CuCl (D) and Y-Cu_2_O/CuCl (E). (F and G) . Potential-dependent population of interfacial water on Cu_2_O/CuCl (F) and Y-Cu_2_O/CuCl (G). (H) Potential-dependent population of K·H_2_O.

Hydrogen sourcing in liquid products was further investigated through ^1^H NMR analysis of ethanol. As evidenced by Fig. S31, the methyl proton resonance at 0.94 parts per million (ppm) exhibited characteristic splitting patterns, which clearly demonstrates that H^+^ is involved in the hydrogenation process of ethanol. To probe hydrogen origin in ethanol products, D_2_O isotopic labeling was employed. Specifically, when H_2_SO_4_ + 0.5 M K_2_SO_4_ in H_2_O electrolyte was employed, a triplet signature was observed, following the *n* + 1 rule (Fig. 5A), whereas 0.5 M K_2_SO_4_ + H_2_SO_4_ in D_2_O electrolyte induced quadruplet splitting (Fig. 5B and C). This isotopic splitting pattern confirmed partial deuterium substitution at the methylene position, demonstrating dual hydrogen origins from both H^+^ and water-derived protons. It suggests that the reason for the increased selectivity of ethanol and decreased selectivity of ethylene is that ethanol requires more water activation to participate in hydrogenation.

The interfacial water structure is very important for the hydrogenation of CO_2_RR, which was probed through ATR-SEIRAS. O–H stretching vibrations within the 3,000 to 3,800 cm^−1^ spectral range (−0.1 to −1.3 V versus RHE) were deconvoluted via Lorentzian fitting. Three distinct bands were resolved at ~3,600, 3,450, and 3,250 cm^−1^, assigned to K**·**H_2_O complexes, weakly hydrogen-bonded water (w-HB-H_2_O), and strongly hydrogen-bonded water (s-HB-H_2_O) [45]. Figure 5D and E shows the distribution of the 3 water compositions of Cu_2_O/CuCl and Y-Cu_2_O/CuCl at different voltages. Comparing the K**·**H_2_O ratios of Cu_2_O/CuCl and Y-Cu_2_O/CuCl in eCO_2_RR (Figs. 5F and 6H and Tables S3 and S4), the K**·**H_2_O ratio of Y-Cu_2_O/CuCl was markedly higher than that of Cu_2_O/CuCl at different voltages. This K**·**H_2_O formation originates from K^+^ cation hydration, where K^+^ attracts oxygen atoms of water to coordinate surrounding water molecules. The markedly elevated K**·**H_2_O ratio in Y-doped systems indicates promoted interfacial water activation, facilitating *H transfer during CO_2_RR. The pathway mechanism for ethanol production generates *CH–COH to *CH–CHOH. H_2_O hydrogenation is kinetically and thermodynamically disadvantaged because the carbon end of the intermediate is spatially blocked by the surface structure. Therefore, the Y site in the catalyst Cu–O–Y unit can appropriately promote the activation of K**·**H_2_O, and the resulting *H can migrate rapidly to accelerate *CH–COH + *H → *CH–CHOH. This compositional shift correlates with enhanced ethanol selectivity, as activated water networks lower kinetic barriers for hydrogenation steps.

## Conclusion

In summary, we reveal a CuCl-mediated synthesis strategy to regulate particle size by leveraging the confinement effect of porous spheres, thereby enhancing intermediate enrichment and improving multi-carbon product selectivity. Y doping in Cu_2_O/CuCl promotes interfacial water participation, modulating catalytically active hydrogen species to enhance ethanol selectivity. For the first time, DEMS and NMR spectroscopy were employed to analyze hydrogen species in liquid and gaseous eCO_2_RR products. We reveal that in acidic electrolytes, the ethylene pathway preferentially undergoes H^+^ hydrogenation, while both H^+^ and *H participate in ethanol formation. Y doping effectively activates interfacial water, supplying additional *H and achieving 65.7% ethanol selectivity with excellent stability. This work provides a new understanding of the design of ethanol catalysts in eCO_2_RR.

## Materials and Methods

### Cu_2_O synthesis

A triblock copolymer (P123, 2.448 g) was dissolved in 120 ml of deionized water, under continuous stirring at room temperature. Separately, 10 ml of 0.2 M copper (II) acetate solution was mixed with 1.5 ml of aqueous ammonia (25% to 28%) to form a homogeneous copper–ammonia complex solution. This solution was then added to the P123 mixture and stirred for 30 min. Subsequently, 20 ml of 0.6 M ascorbic acid solution was introduced and stirred vigorously for an additional 30 min. The resulting product was collected by centrifugation, followed by 5 washing cycles with deionized water and ethanol to remove impurities.

### Cu_2_O/CuCl synthesis

The procedure remained identical to that of Cu_2_O synthesis, except that 0.2 M copper (II) acetate was replaced with 0.2 M copper (II) chloride.

### Y-Cu_2_O/CuCl synthesis

For yttrium incorporation, 0.006 mol of yttrium nitrate was added to 10 ml of 0.2 M copper(II) chloride solution prior to mixing with ammonia. The subsequent steps followed the same protocol as described for Cu_2_O/CuCl synthesis.

## Data Availability

Detailed data are provided in the main text and in the Supplementary Materials. The additional data used to support the findings of this study are available from the corresponding authors upon request.
