# Supplementary material for: Confinement Effect and Hydrogen Species Modulation toward Enhanced Electrochemical CO2 Reduction to Ethanol
Source: Research (Wash D C). 2025 Jul 31;8:0796. doi: 10.34133/research.0796 (PMC12311302; doi:10.34133/research.0796)
Supplement: Supplementary 1 — Supplementary Text Figs. S1 to S31 Tables S1 to S4 [file research.0796.f1.docx]

Supplementary Information for

**Confinement Effect and Hydrogen Species Modulation toward Enhanced Electrochemical CO_2_ Reduction to Ethanol**

Yuting Zhu^1^, Jiamin Zhu^1^, Huizhi Li^1^, Shuhui Li^1^, Yue Zhai^1^, Shao-Wen Xu^1^, Shanshan Wu^1^, Yuan Chen^1^, Li An^1*^, Pinxian Xi^1*^, Chun-Hua Yan^1,2^

^1^State Key Laboratory of Applied Organic Chemistry, Frontiers Science Center for Rare Isotopes, College of Chemistry and Chemical Engineering, Lanzhou University, China

^2^Beijing National Laboratory for Molecular Sciences, State Key Laboratory of Rare Earth Materials Chemistry and Applications, PKU-HKU Joint Laboratory in Rare Earth Materials and Bioinorganic Chemistry, College of Chemistry and Molecular Engineering, Peking University, China

**Experiment section**

# Physicochemical Characterizations

XRD measurements were carried out on a Rigaku D/Max-2400 diffractometer with Cu Kα radiation (*λ* = 0.1542 nm) under a constant voltage of 40 kV. TEM, HRTEM pictures, and EDX mapping of samples were obtained on a Tecnai G2 F30 filed emission TEM. Atomic-scale STEM images were recorded on a probe aberration corrected STEM (Cubed Titan G2 60-300, FEI, USA) operated at 300 kV. Transmission XAS measurements were performed on a laboratory device (easyXAFS300+, easyXAFS LLC), which is based on Rowland circle geometries with spherically bent crystal analyzers (SBCA) and a silicon drift detector. Si(5,5,3) was used for Cu K-edge measurement. The powder samples were thoroughly ground and mixed with Boron Nitride using an agate mortar and pestle and pressed into Ø = 10 mm pellets. The pressed pellets were then sandwiched by Kapton tapes. X-ray photoelectron spectroscopy (XPS) analysis was made with a Kratos Axis Supra device a VG ESCALAB 220I-XL device. All XPS spectra were corrected using C1s line at 284.8 eV. Inductively coupled plasma optical emission spectrometry (ICP-OES) analyses were performed on a Plasma Quant PQ9000 ICP spectrometer.

# Preparation of the Working Electrode

The working electrode was prepared by mixing 10 mg of catalyst, 2.5 mg of carbon black, and 2 mg of PVDF, followed by adding 3–4 drops of N-methylpyrrolidone (NMP) and grinding the mixture for 10 minutes to form a homogeneous slurry, which was then uniformly coated onto carbon paper using a brush and dried overnight in a vacuum oven.

# Electrochemical CO_2_RR Measurements

The electrochemical CO_2_ reduction experiments were conducted using a CHI760E electrochemical workstation. A flow cell configuration was employed, consisting of a platinum foil anode, an Ag/AgCl reference electrode and a custom-prepared working electrode as the cathode. The anolyte and catholyte compartments were separated by a Nafion 117 cation-exchange membrane. The catholyte comprised 0.5 M K_2_SO_4_ solution adjusted to pH = 3 using H_2_SO_4_. Before electrolysis, CO_2_ gas was continuously purged into the cathodic chamber at a flow rate of 20 mL min⁻¹ for 30 min to saturate the electrolyte, and this flow was maintained during the reaction.

# Product Analysis Methods

The gaseous products of the electrochemical CO_2_ reduction reaction (CO_2_RR) were analyzed using online gas chromatography (GC). Gas species were identified by comparing their retention times with those of known standards, while their quantities were quantified using calibration curves derived from peak areas. Specifically, hydrogen (H_2_) and carbon monoxide (CO) were detected using a thermal conductivity detector (TCD), and hydrocarbon products (e.g., CH_4_ and C_2_H_4_) were analyzed with a flame ionization detector (FID).

For liquid products, nuclear magnetic resonance (^1^H NMR) spectroscopy was employed. A mixture of 0.5 mL post-electrolysis electrolyte (collected after constant-current electrolysis for a specified duration), 0.1 mL deuterated water (D_2_O), and 0.05 μL dimethyl sulfoxide (DMSO, as an internal standard) was prepared for analysis. Quantification of liquid products (e.g., formate and alcohols) was achieved by integrating peak areas relative to the DMSO reference and applying pre-established calibration curves.

# Faradaic Efficiency Calculation in eCO_2_RR

The Faradaic efficiency (FE) for a specific product in CO_2_ reduction reaction (CO_2_RR) is calculated as follows：

Definitions:

n: Number of electrons required per mole of product (e.g., 2 for CO, 12 for C_2_H_4_)

F: Faraday constant (96,485 C mol^−1^)

m: Moles of product quantified (mol)

I: Total current (A)

t: Electrolysis time (s)

# Calculation of Single-Pass Carbon Efficiency in eCO_2_RR：

Single-Pass Carbon Efficiency (SPCE) quantifies the fraction of carbon atoms from the input CO_2_ that are converted into a specific target product during a single pass through the electrochemical reactor. SPCE is calculated as follows:

1. Measure input CO_2_:

(Assuming ideal gas: P=pressure(atm), R=8.314 J/(mol·K), T=temperature (K))

1. Quantify target product:

Use analytical techniques (GC, NMR) to measure the molar yield of the carbon-based product. Multiply by the number of carbon atoms per product molecule:

1. Calculate SPCE:

# RRDE measurements

The evolution of pH at the disk electrode surface can be monitored through changes in the open circuit potential (OCP) of an IrOx-modified ring electrode. The IrOx layer was electrodeposited onto the Pt ring of a rotating ring-disk electrode (RRDE) via cyclic voltammetry (CV) under the following conditions: a potential range of -0.45 to 0.75 V vs. Ag/AgCl, a scan rate of 1.0 V s⁻¹, 300 cycles starting in the negative direction, and an Ar-saturated electrolyte prepared according to reported methods. Subsequently, the OCP (EOC) of the IrOx-modified ring electrode was measured in 0.5 M NaCl electrolyte with pH adjusted by incremental NaOH addition. A linear calibration curve between EOC and pH was established (Figure S3), yielding the relationship pHᵣᵢₙg = -(EOC - b)/a, where a and b represent the slope and intercept from the linear fit. For real-time pH monitoring during the oxygen evolution reaction (OER), the disk electrode was loaded with 10 μL of catalyst ink and tested in Ar-saturated alkaline saline electrolyte. Linear sweep voltammetry (LSV) was performed on the disk at 1 mV s⁻¹ while simultaneously recording the EOC of the IrOx ring. The ring pH (pH_ring_) was derived from equation (1), and the disk surface pH (pH_disk_) was calculated using equation (2): Cₕ⁺,_ring_ - Cₒₕ⁻,_ring_ = Nᴅ(Cₕ⁺,_disk_ - Cₒₕ⁻,_disk_) + (1 - Nᴅ)(Cₕ⁺,_bulk_ - Cₒₕ⁻,_bulk_), where Cₘ,ₙ denotes species concentrations at the ring, disk, or bulk, and Nᴅ = 0.36 represents the predefined detection efficiency.

# In situ EQCM measurements

The EQCM measurements were conducted using an EQCM 15M system (Gamry, USA) integrated with a temperature-controlled eCell microchamber (Hettich, Germany). A 9.12 MHz ± 50 kHz AT-cut quartz crystal served as the substrate, where Au polycrystalline thin-film catalysts deposited by spin coating functioned as the working electrode. This electrode assembly was installed in a three-compartment electrochemical cell, with a Hg/HgO reference electrode and Pt wire counter electrode completing the setup. Frequency changes were monitored via a Q-sensor analyzer (QE 401) coupled to a QEC 401 electrochemistry module. Potential-dependent frequency transitions were acquired through steady-state polarization and cyclic voltammetry, with data collection managed by Gamry Resonator software. For mass quantification, the Sauerbrey equation () was applied to parallel frequency (fp) data, where Δf represents frequency variation, C_f_ is the theoretical correction factor (56.6 Hz·cm²/μg for 5 MHz chips), and m denotes surface mass changes.

# In situ Raman measurement

Raman spectroscopy was performed using a LabRAM HR Evolution system equipped with a 532 nm excitation source. *In situ* electrochemical Raman measurements were conducted in 1 M KOH seawater electrolyte using a specialized cell (031-2H), featuring a saturated Ag/AgCl reference electrode and Pt ring counter electrode, while applying controlled constant voltages to monitor real-time surface chemical composition and structural evolution of materials. Surface characterization was achieved by maintaining specific applied potentials for 10-minute intervals during *in situ* spectral acquisition.

# In-situ ATR-SEIRAS spectroscopy:

In-situ ATR-IR spectroscopy measurements were performed in 1.0 M KOH electrolyte using a saturated Ag/AgCl reference electrode and a platinum ring counter electrode. Surface chemical composition and structural information of the materials were acquired through *in-situ* electrochemical characterization at specific applied potentials held for 10-minute intervals.

**DEMS measurements:**

Differential electrochemical mass spectrometry (DEMS) measurements were performed using a QAS 100 system (Linglu Instruments, Shanghai) to monitor D_2_O-labeled adsorbed hydrogen and track hydrogen ion (H⁺) participation via isotopic signatures during the eCO_2_ reduction reaction (eCO_2_RR). To more accurately monitor the isotopic distribution in the eCO_2_RR process, effluent gases from the flow cell were introduced into the DEMS for continuous 180-second intervals. Flow cell testing conditions are detailed in the "Electrochemical CO_2_RR Measurements" section. Isotopic tracing was implemented in D_2_O-based electrolyte containing H_2_SO₄ and 0.5 M K_2_SO₄ to deuterium-label water species. Gas-phase products were analyzed in real time by configuring DEMS detection channels for m/z = 2 (H_2_), 3 (HD), 4 (D_2_) and parallel monitoring of m/z = 28–32 for ethylene isotopologues (C_2_H_4_, C_2_H_3_D, C_2_H_2_D_2_, C_2_HD_3_, C_2_D_4_). A three-electrode setup with Ag/AgCl reference and Pt wire counter electrodes was maintained throughout the experiments.

**

**

**Supplementary Fig. S1** UV-Vis of mixed solutions before the addition of ascorbic acid in the synthesis of Cu_2_O/CuCl.





**Supplementary Fig. S2** XRD pattern of CuCl.

**Supplementary Fig. S3** The XRD patterns of Cu_2_O/CuCl.





**Supplementary Fig. S4** UV-Vis of mixed solutions before the addition of ascorbic acid in the synthesis of Cu_2_O.

**Supplementary Fig. S5** The XRD patterns of Cu_2_O.





**Supplementary Fig. S6** EPR of Cu_2_O and Cu_2_O/CuCl.

**Supplementary Fig. S7** FWHM of Cu_2_O and Cu_2_O/CuCl XRD pattern.


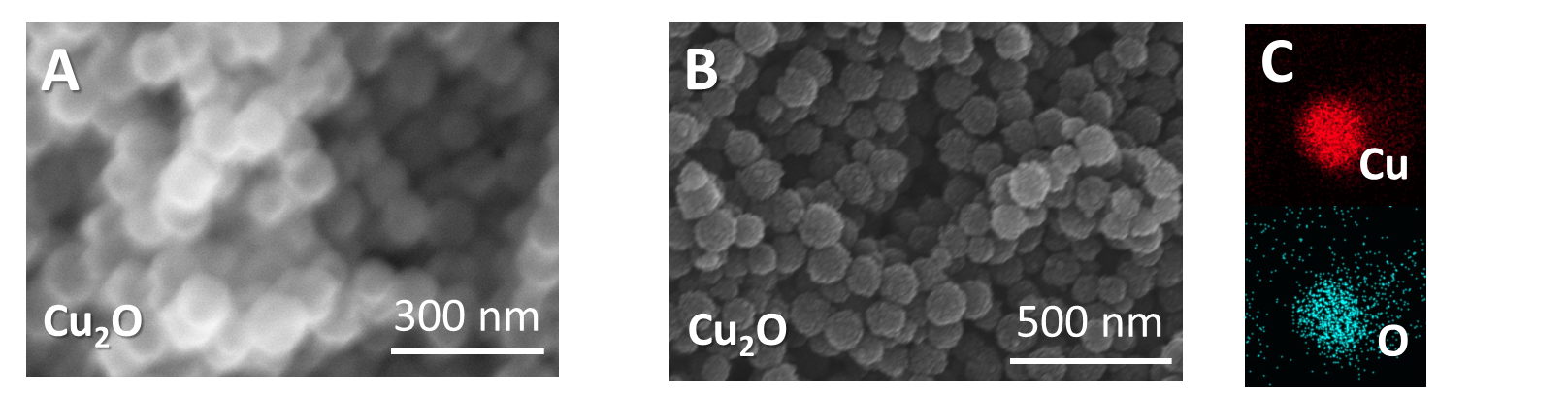


**Supplementary Fig. S8** The SEM image of Cu_2_O at 300 nm (A) and 500 nm (B), EDS mapping of Cu_2_O.


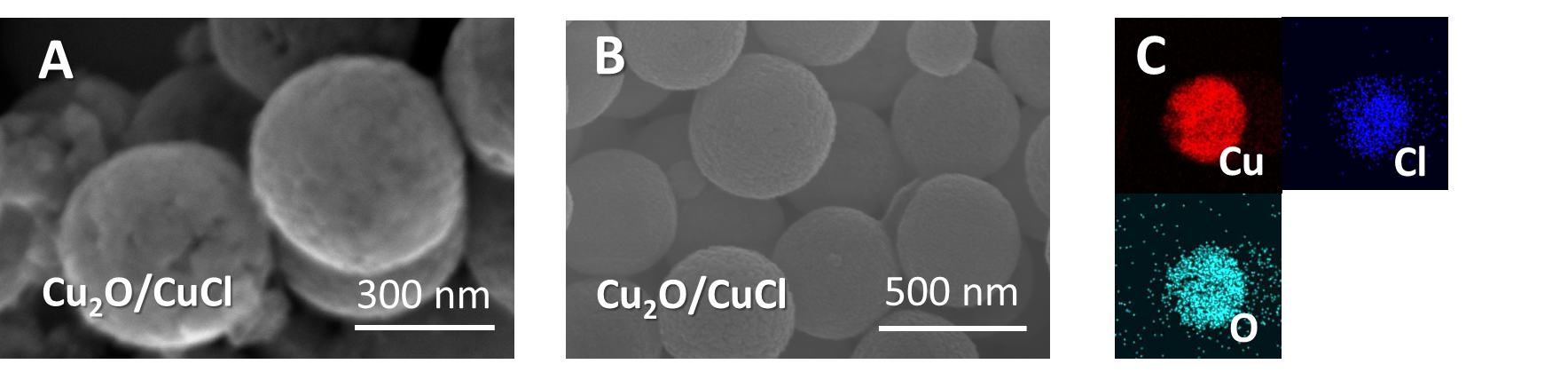


**Supplementary Fig. S9** The SEM image of Cu_2_O/CuCl at 300 nm (A) and 500 nm (B), EDS mapping of Cu_2_O/CuCl.

**

**

**Supplementary Fig. S10** HRTEM image of Cu_2_O (111) plane.





**Supplementary Fig. S11** EDX mapping of Cu_2_O/CuCl.


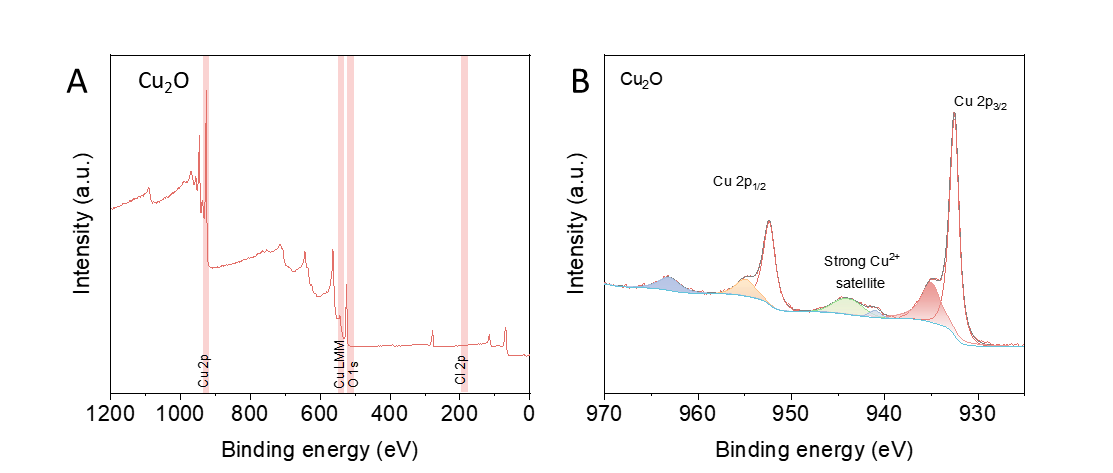


**Supplementary Fig. S12** (A) Cu_2_O XPS spectrum, (B) Cu 2p XPS spectrum of Cu_2_O.


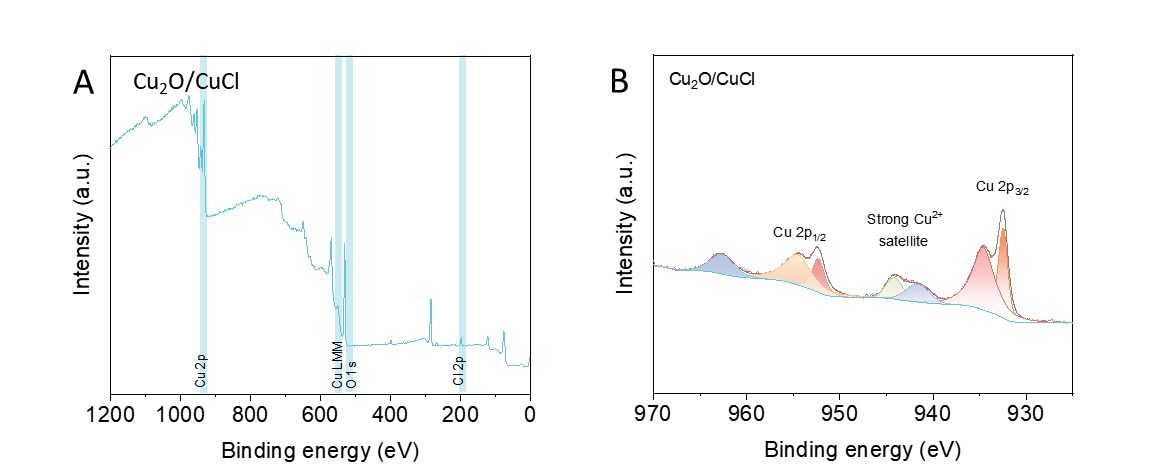


**Supplementary Fig. S13** (A) Cu_2_O XPS spectrum, (B) Cu 2p XPS spectrum of Cu_2_O/CuCl.


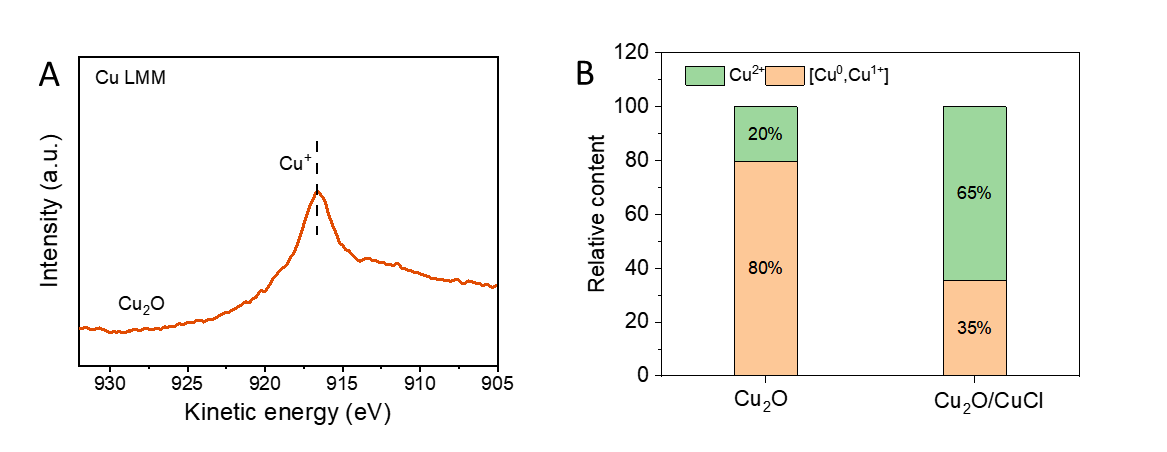


**Supplementary Fig. S14** (A) Cu Auger LMM spectrum of Cu_2_O, (B) Relative amount of (Cu^0^,Cu^1+^) in Cu_2_O and Cu_2_O/CuCl catalysts from Cu 2p_3/2_ XPS.

**

**

**Supplementary Fig. S15** Mott-Schottky plot of Cu_2_O(A) and Cu_2_O/CuCl(B).


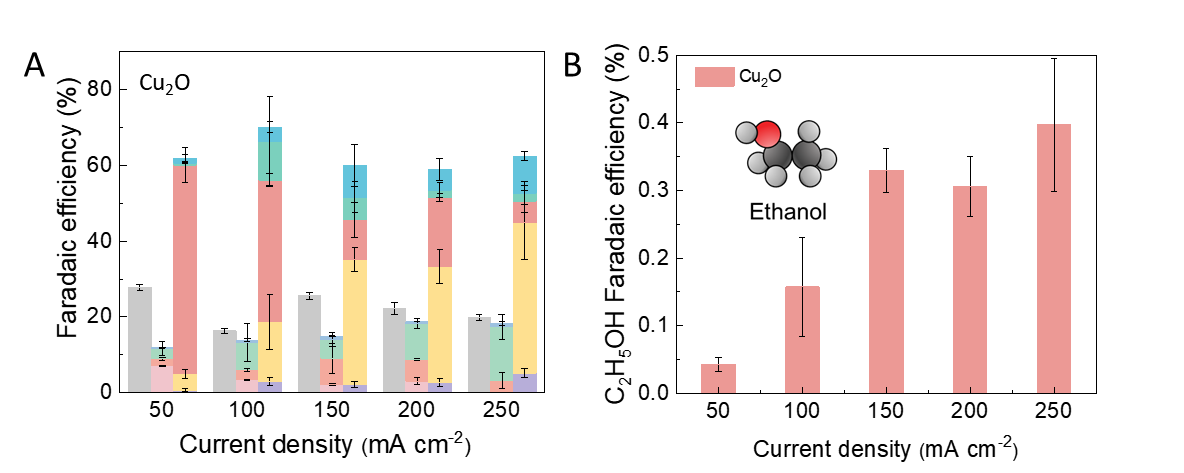


**Supplementary Fig. S16** (A) the Faradaic efficiclency of Cu_2_O, (B) the C_2+_ Faradaic efficiclency of Cu_2_O.


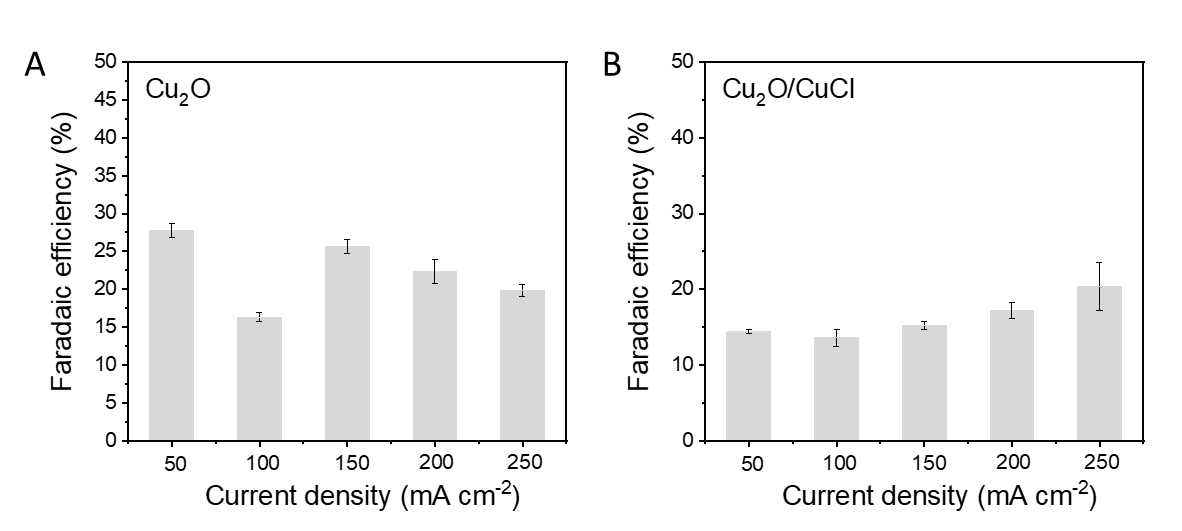


**Supplementary Fig. S17** the H_2_ Faradaic efficiclency of Cu_2_O (A) and Cu_2_O/CuCl (B).


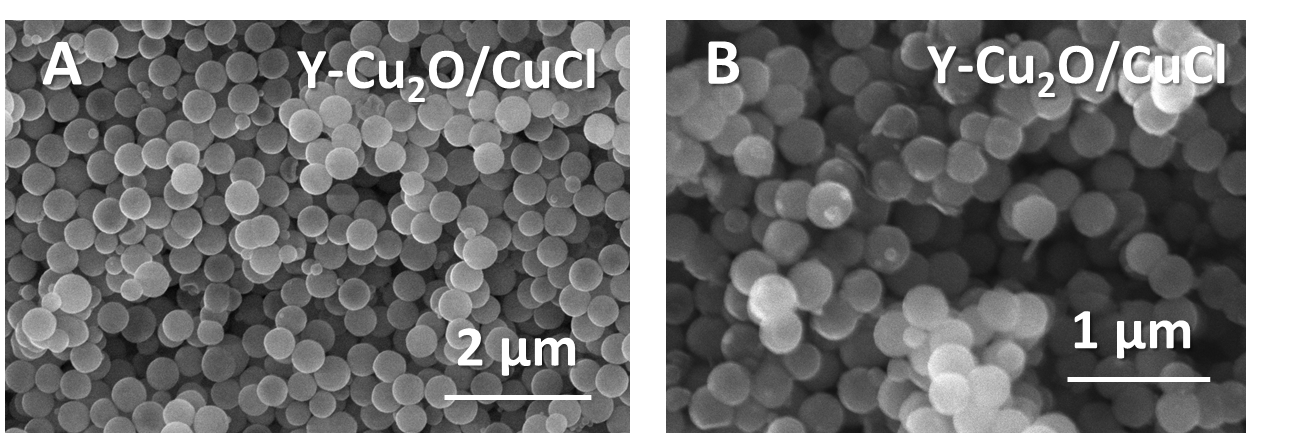


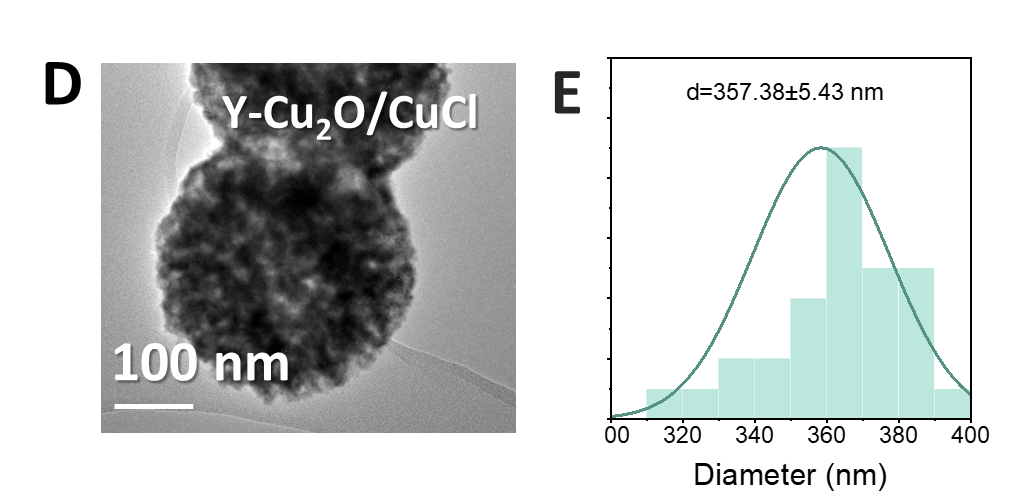


**Supplementary Fig. S18** The SEM image of Y-Cu_2_O/CuCl at 2 µm (A), 1 µm (B) and 300nm (C). (D) The average diameter of Y-Cu_2_O/CuCl. (E) The TEM image of Y-Cu_2_O/CuCl. (F) spherical aberration corrected dark-field (AC-DF) TEM image of Y-Cu_2_O/CuCl at 100nm.


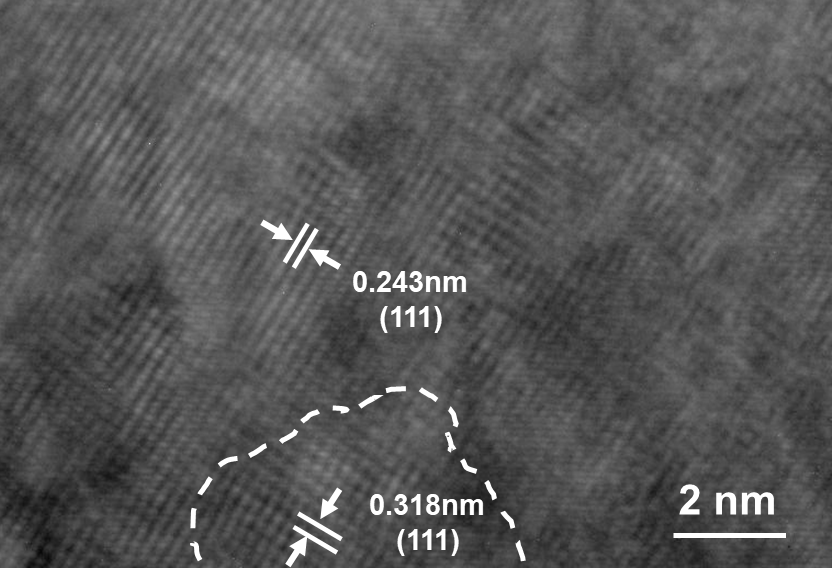


**Supplementary Fig. S19** HRTEM image showing representative interface profiles within Cu_2_O/CuCl


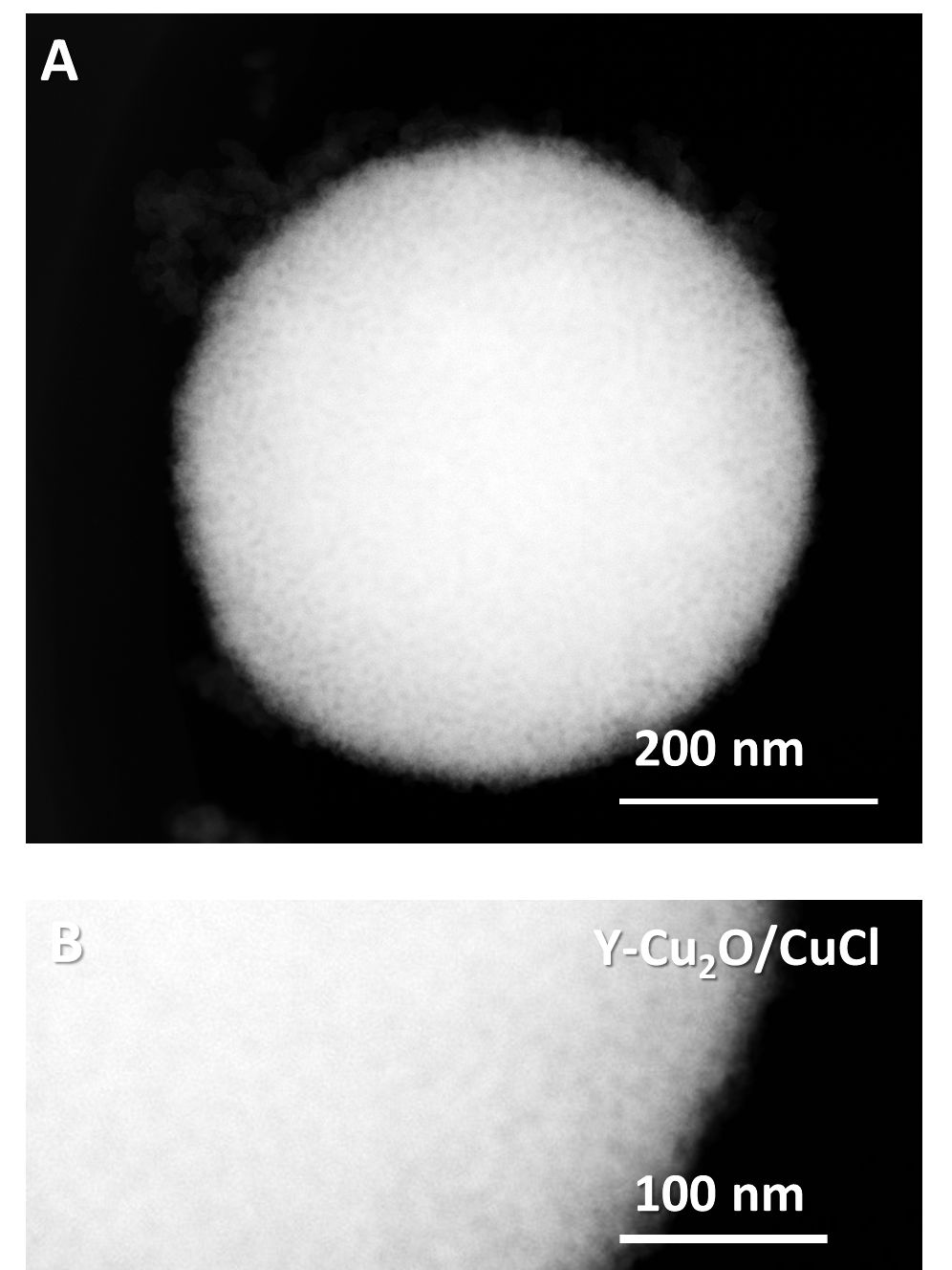


**Supplementary Fig. S20** Spherical aberration corrected dark-field (AC-DF) TEM image of Y-Cu2O/CuCl at 200nm (A) and100 nm (B).

**
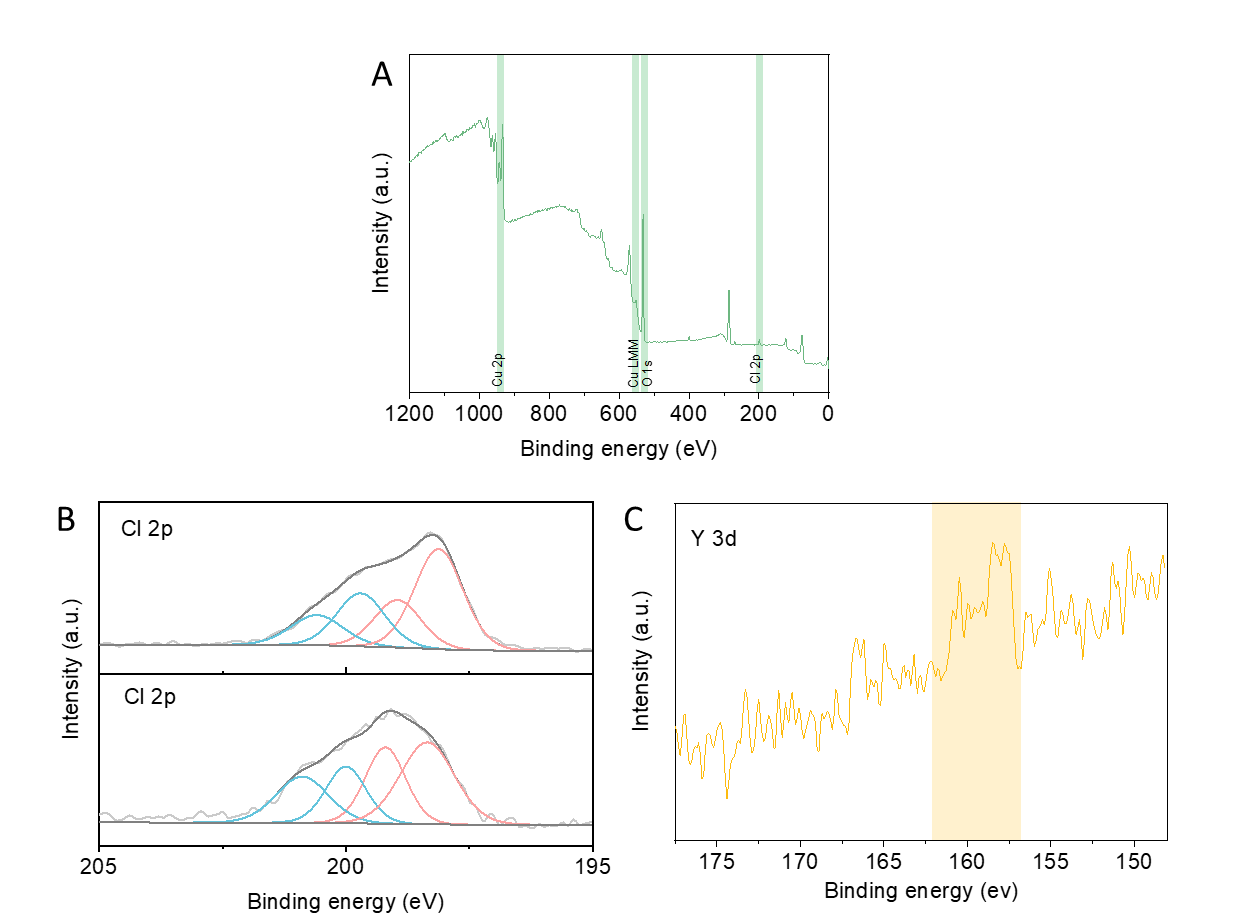
**

**Supplementary Fig. S21** (A) Y-Cu_2_O/CuCl XPS spectrum, (B) Cl 2p XPS spectrum of Cu_2_O/CuCl and Y-Cu_2_O/CuCl, (C) Y 3d XPS spectrum of Y-Cu_2_O/CuCl.


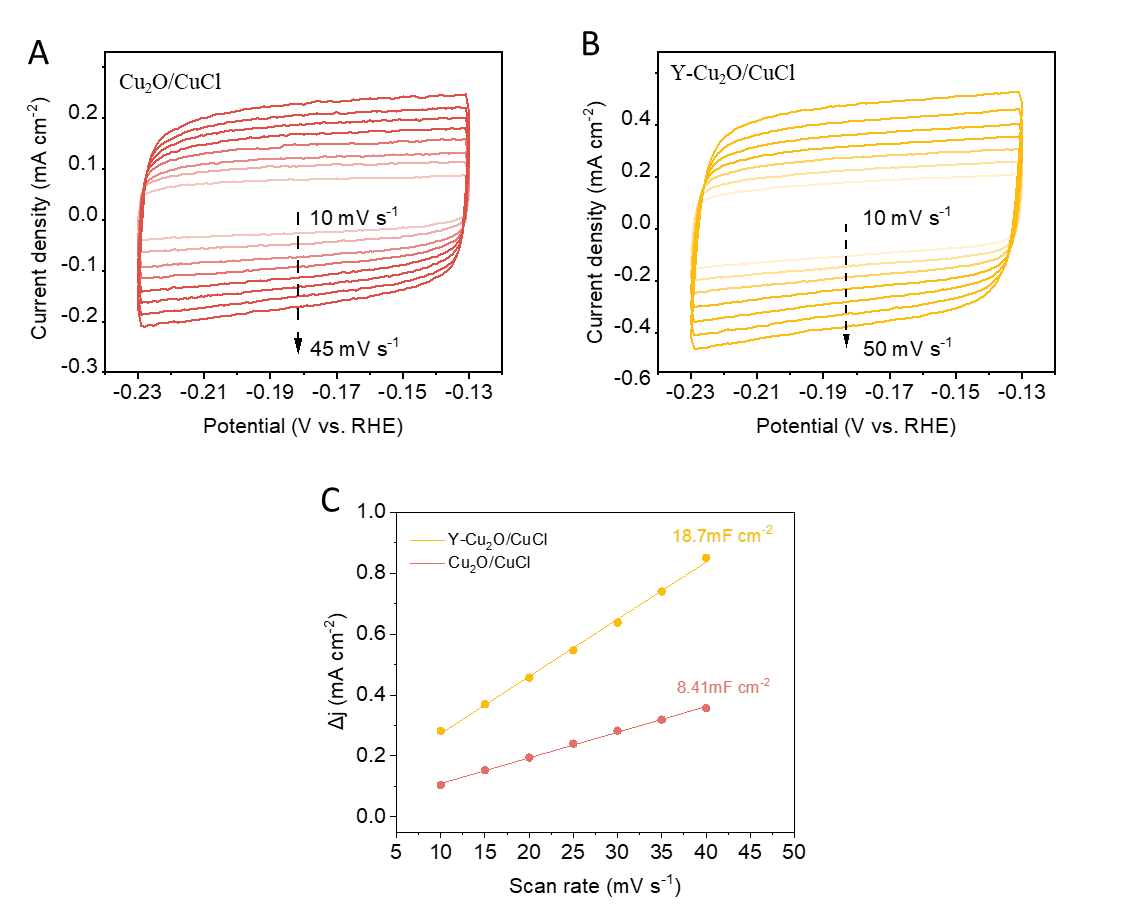


**Supplementary Fig. S22** (A,B) CV curves for Cu_2_O/CuCl(A) and Y-Cu_2_O/CuCl(B) in the region of 0.13~0.23 V vs. RHE with various scan rates for CO_2_OR. (C) C_dl_ of Cu_2_O/CuCl and Y-Cu_2_O/CuCl.


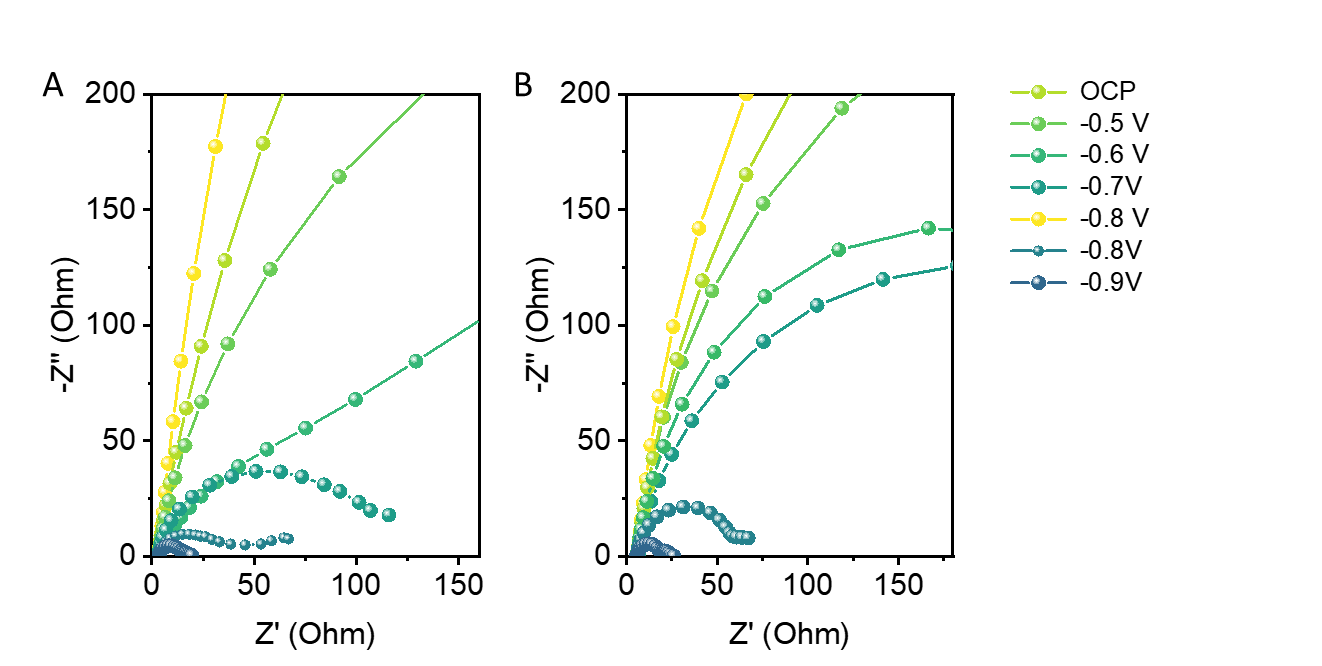


**Supplementary Fig. S23** (A,B) Nyquist plots of Cu_2_O/CuCl(A) and Y-Cu_2_O/CuCl(B) fitted at different potentials.

**
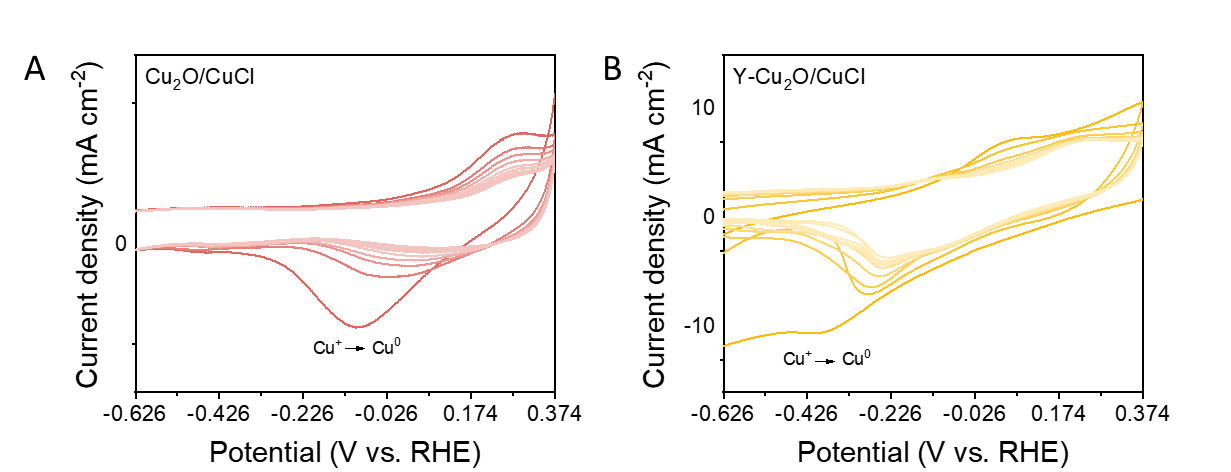
**

**Supplementary Fig. S24** (A,B) CV tests of Cu_2_O/CuCl(A) and Y-Cu_2_O/CuCl(B).


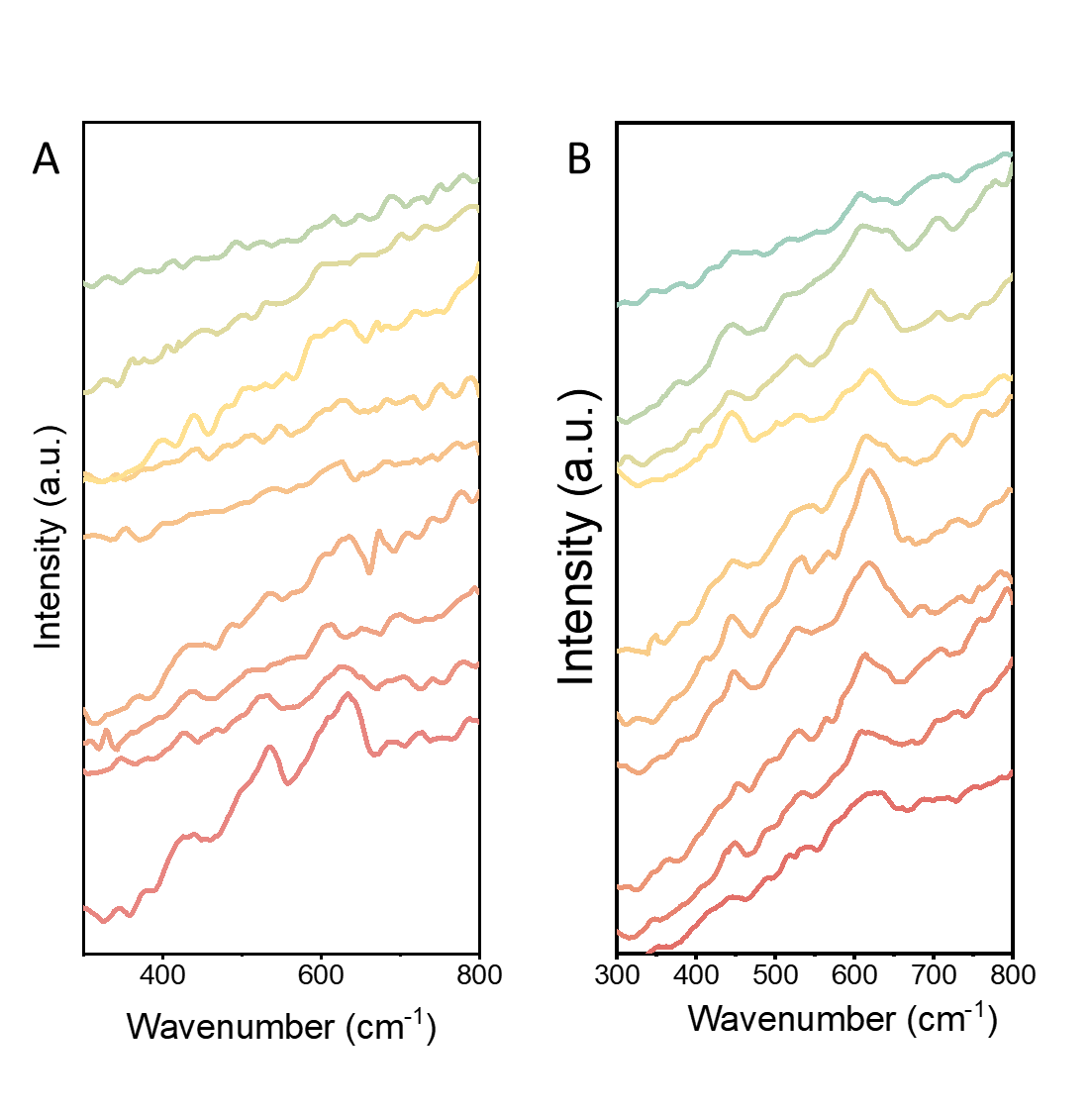


**Supplementary Fig. S25** (A,B)in situ surface-enhanced Raman spectroscopy of Cu_2_O/CuCl (A) and Y-Cu_2_O/CuCl (B).

**Supplementary Fig. S26** XRD pattern for Y-Cu_2_O/CuCl after electrolysis 30 min at 200 mA cm^-2^.

**Supplementary Fig. S27** Chloride ion concentration in Y-Cu_2_O/CuCl as a function of time.


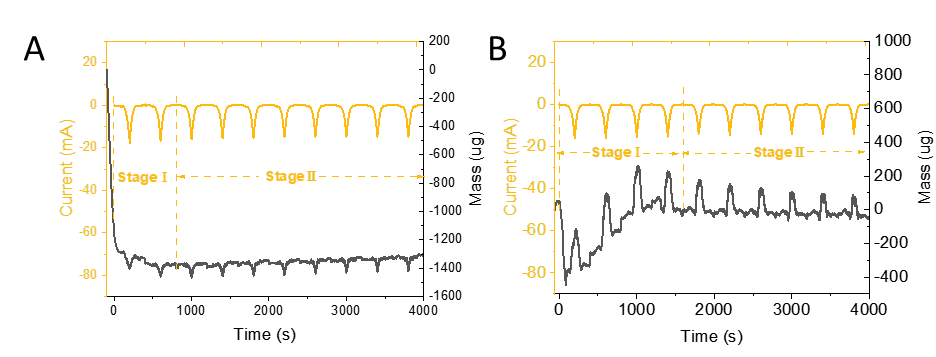


**Supplementary Fig. S28** Plot of mass change during CV scanning of Cu_2_O/CuCl (A) and Y-Cu_2_O/CuCl (B).


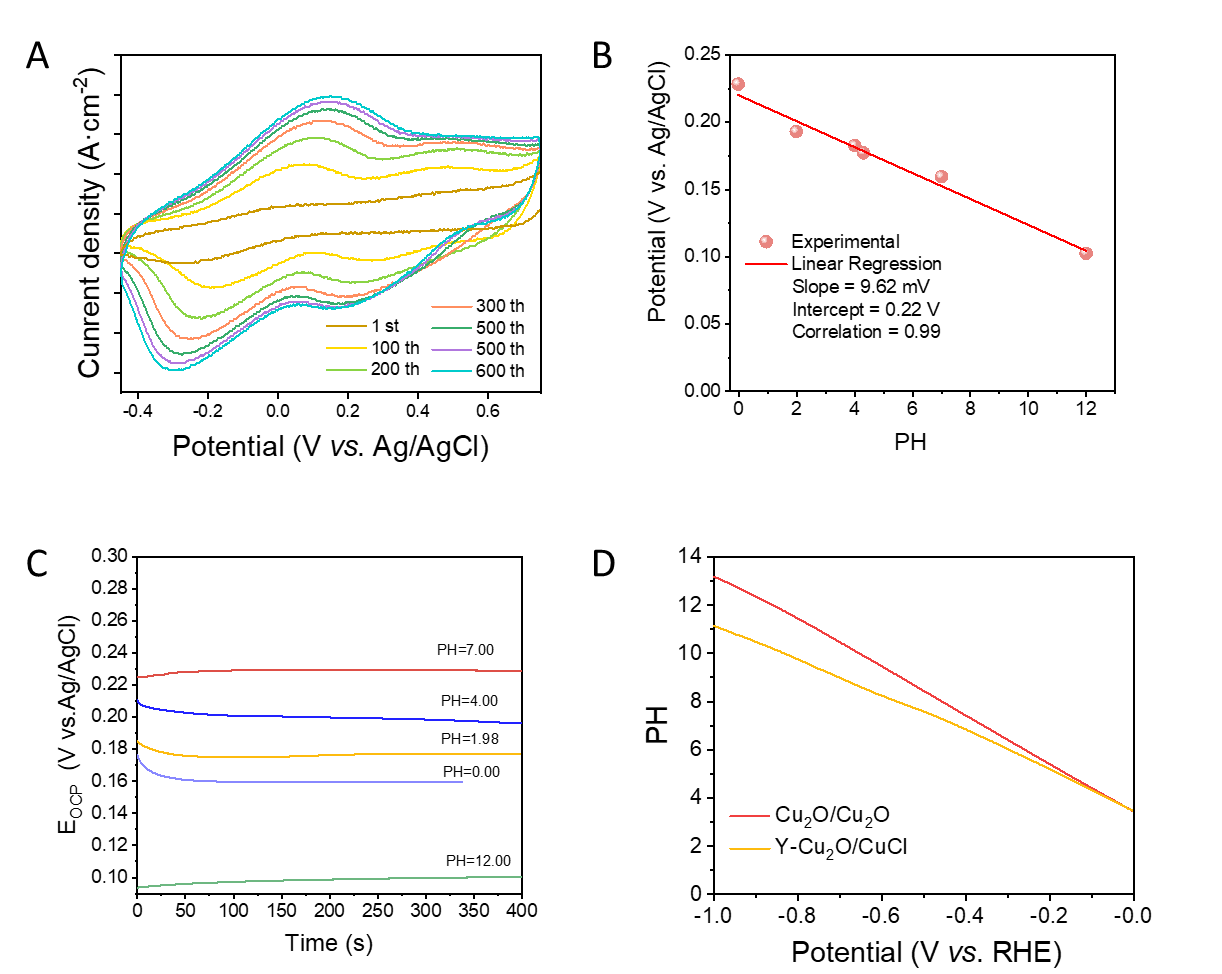


**Supplementary Fig. S29** (A) CV curves for IrO_x_ deposition on ring electrodes. (B) Fitted plots of the relationship between different pH values and electrode OCP. (C) Plots of the OCP of the electrode as a function of time in solutions of different PH. (D) Plot of pH versus potential on the surface of Cu_2_O/CuCl and Y-Cu_2_O/CuCl.


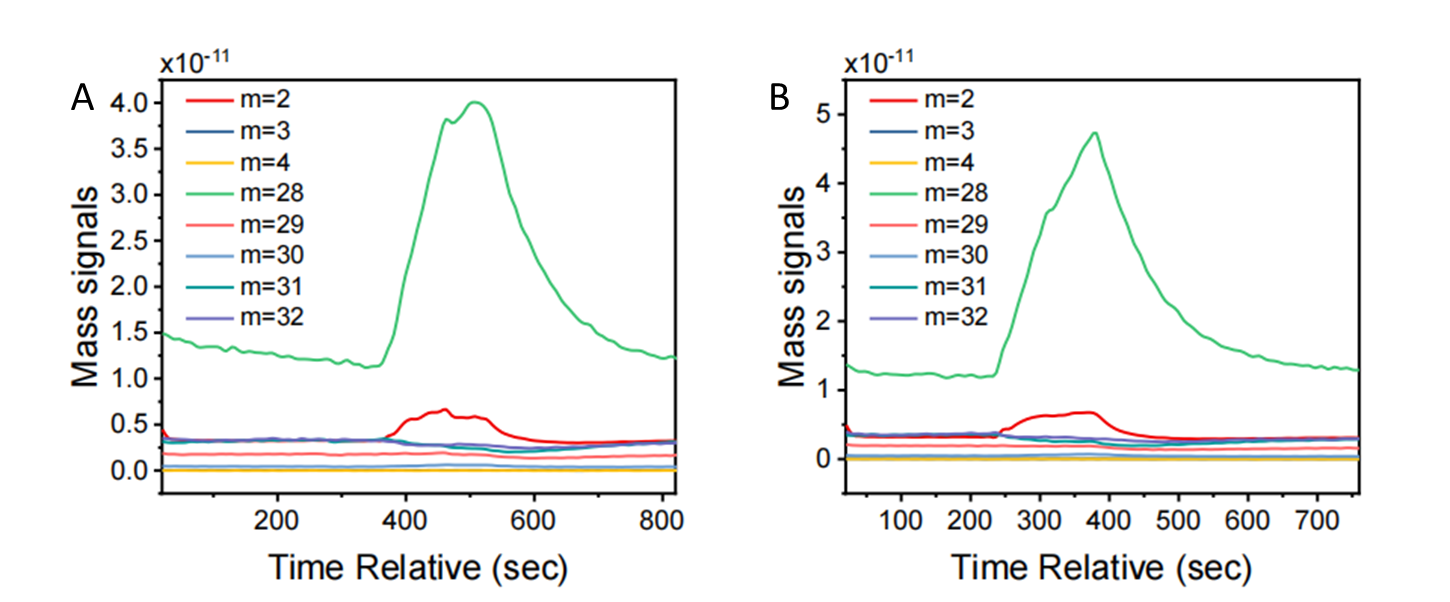


**Supplementary Fig. S30** DEMS measurements of Cu_2_O/CuCl (A) and Y-Cu_2_O/CuCl (B) in 0.5 M K_2_SO_4_ + H_2_SO_4_(PH=3) with H_2_O.

**Supplementary Fig. S31** ¹H NMR spectroscopy on the liquid-phase products after electrolysis of Y-Cu_2_O/CuCl in aqueous 0.5 M K_2_SO_4_ + H_2_SO_4_ electrolyte with D_2_O (pH = 3)

**Supplementary Table S1** Table of ICP test results for Y-Cu₂O/CuCl.

| **Catalysts** | **Cu(mg L^−1^)** | **Y (mg L^−1^)** | **Y (*ω* %)** |
| --- | --- | --- | --- |
| Y-Cu_2_O/CuCl | 37.93 | 0.0438 | - |
| Y-Cu_2_O/CuCl | 37.98 | 0.0438 | - |
| Average | 37.955 | 0.0438 | 0.115 |

**Supplementary Table S2** Activity and Stability performance

of Y-Cu_2_O/CuCl compared to other catalysts.

| **Electrocatalysts** | **Current density (mA cm^-2^)** | **FE_ethanol_ (%)** | **Stability time (h)** | **Electrolytic cell type** | **Electrolyte** | **References** |
| --- | --- | --- | --- | --- | --- | --- |
| **Y-Cu_2_O/CuCl** | **0.25** | **57** | **65** | **flow cell** | **0.5 mol/L K_2_SO_4_ (PH=3)** | **This work** |
| LA Cu_2_O | 0.4 | 25 | 2 | flow cell | 2 mol/L KCl | 29 |
| Cu^δ+^ | 0.4 | 48.1 | 10 | MEA | 0.02 mol/L KHCO_3_ | 30 |
| BaO/Cu | 0.4 | 45 | 20 | flow cell | 1 mol/L KOH | 31 |
| F-Cu | 1.6 | 15 | 40 | flow cell | 2 mol/L KOH | 32 |
| FeTPP[Cl]/Cu | 0.124 | 41 | 2 | flow cell | 1 mol/L KHCO_3_ | 33 |
| Ce(OH)_x_-Cu | 0.128 | 43 | 6 | flow cell | 1 mol/L KOH | 34 |
| [CuO_x_@C](mailto:CuOx@C) | 0.164 | 45 | 50 | flow cell | 1 mol/L KOH | 35 |
| Ag/Cu_2_O | 0.32 | 45 | 6 | flow cell | 1 mol/L KOH | 36 |

**Supplementary Table S3** Population of S-HB-H_2_O, W-HB-H_2_O and K·H_2_O on the Cu_2_O/CuCl.

| **Potential**  **(V vs. RHE)** | **Population of**  **S-HB-H_2_O (%)** | **Population of**  **W-HB-H_2_O (%)** | **Population of**  **K·H_2_O (%)** |
| --- | --- | --- | --- |
| -0.1 | 46.80029 | 38.7773 | 14.42241 |
| -0.5 | 50.07883 | 37.02093 | 12.90024 |
| -0.9 | 55.05767 | 41.09004 | 3.85229 |
| -1.3 | 47.44874 | 38.65264 | 13.89862 |

**Supplementary Table S4** Population of S-HB-H_2_O, W-HB-H_2_O and K·H_2_O on the Y-Cu_2_O/CuCl.

| **Potential**  **(V vs. RHE)** | **Population of**  **S-HB-H_2_O (%)** | **Population of**  **W-HB-H_2_O (%)** | **Population of**  **K·H_2_O (%)** |
| --- | --- | --- | --- |
| -0.1 | 28.09012 | 46.2137 | 25.69618 |
| -0.5 | 34.832 | 44.26542 | 20.90259 |
| -0.9 | 34.38347 | 44.29451 | 21.32202 |
| -1.3 | 41.80625 | 34.95421 | 23.23954 |
